# The application of ROBINS-I guidance in systematic reviews of non-randomised studies: A descriptive study

**DOI:** 10.1017/rsm.2025.10048

**Published:** 2025-10-22

**Authors:** Zipporah Iheozor-Ejiofor, Jelena Savović, Russell J. Bowater, Julian P.T. Higgins

**Affiliations:** Population Health Sciences, Bristol Medical School, University of Bristol, Bristol, UK

**Keywords:** bias, non-randomised, observational, ROBINS-I

## Abstract

The ROBINS-I tool is a commonly used tool to assess risk of bias in non-randomised studies of interventions (NRSI) included in systematic reviews. The reporting of ROBINS-I results is important for decision-makers using systematic reviews to understand the weaknesses of the evidence. In particular, systematic review authors should apply the tool according to the guidance provided. This study aims to describe how ROBINS-I guidance is currently applied by review authors. In January 2023, we undertook a citation search and screened titles and abstracts of records published in the previous 6 months. We included systematic reviews of non-randomised studies of intervention where ROBINS-I had been used for risk-of-bias assessment. Based on 10 criteria, we summarised the diverse ways in which reviews deviated from or reported the use of ROBINS-I. In total, 492 reviews met our inclusion criteria. Only one review met all the expectations of the ROBINS-I guidance. A small proportion of reviews deviated from the seven standard domains (3%), judgements (13%), or in other ways (1%). Of the 476 (97%) reviews that reported some ROBINS-I results, only 57 (12%) reviews reported ROBINS-I results at the outcome level compared with 203 reviews that reported ROBINS-I results at the study level alone. Most systematic reviews of NRSIs do not fully apply the ROBINS-I guidance. This raises concerns around the validity of the ROBINS-I results reported and the use of the evidence from these reviews in decision-making.

## Highlights

### What is already known?

Systematic reviews of NRSIs are used to answer research questions where randomised controlled trials are not available or suitable. The use of systematic review evidence in decision-making requires an understanding of the weaknesses in the evidence. The ROBINS-I tool was developed for this purpose and is very widely used.

### What is new?

Our examination of the implementation of ROBINS-I in 492 reviews revealed that only one review fully adhered to ROBINS-I as intended by the developers. Systematic review authors rarely report outcome-specific ROBINS-I results.

### Potential impact for RSM readers

It is concerning that ROBINS-I results reported in systematic reviews do not fully comply with the guidance. Of particular concern is the failure to report ROBINS-I results at the outcome level, which may have implications for the usability of systematic review evidence from NRSIs in decision-making.

## 1 Background

Systematic reviews are widely used to inform clinical and public health decisions and in the development of guidelines.^1^^,^ ^2^ They are regarded as comprehensive, unbiased sources of evidence due to the rigorous and transparent processes involved in producing them. A key step, which ensures that systematic reviews meet the standards to which they are held, is the assessment of risk of bias in the individual studies that contribute evidence. This is particularly important in systematic reviews of non-randomised or observational studies because such studies are more prone to bias than randomised trials. Different types of risk-of-bias tools are used for assessing different study designs.^3^ One of the most commonly used tools for assessing non-randomised studies of interventions (NRSI)—which is recommended by the Cochrane Collaboration—is the ‘risk of bias in non-randomised studies of interventions’ (ROBINS-I) tool.

ROBINS-I is a domain-based assessment tool for comparative intervention studies that do not use randomisation in allocating study participants to interventions. The main version targets follow-up studies such as observational cohort studies and non-randomised experimental studies,^4^ and versions are in development for case–control studies and other designs. ROBINS-I assesses study results in relation to seven bias domains: bias due to confounding, selection of participants into the studies, classification of interventions, deviation from intended intervention, missing data, measurement of outcome, and selection of the reported result. Within each bias domain is a series of signalling questions, eliciting information about the study relevant to potential biases in that domain. Considerations are combined across the seven domains to reach a judgement of low, moderate, serious, or critical risk of bias overall.

The introduction of the ROBINS-I tool has led to notable changes in evidence-based medicine methods. The original paper describing ROBINS-I has been very highly cited (over 10,000 up to the end of 2023 according to Google Scholar), and the tool has become widely used in systematic reviews.^5^ The GRADE (Grading of Recommendations Assessment, Development, and Evaluations) working group has changed its procedure for when ROBINS-I is used, such that NRSIs are no longer mapped to a starting point of ‘Low certainty’ because of their inherent risk of bias, but instead are judged on their merits using the ROBINS-I tool.^6^^,^ ^7^

Application of the ROBINS-I tool is a detailed process. Despite extensive guidance, it is widely misapplied in systematic reviews of NRSI.^8^ This raises concerns around the trustworthiness of the results of such systematic reviews and whether their assessments reflect the true strengths and limitations of the evidence. Given the importance of risk-of-bias assessments to systematic reviews, deviation from the ROBINS-I guidance may undermine the quality of the systematic review evidence and its conclusions. Furthermore, distorted assessments of the validity of studies could potentially be misleading for evidence users. This review seeks to provide a description of the extent of adherence to the ROBINS-I guidance in systematic reviews of NRSIs and highlight whether further improvements to the implementation of the ROBINS-I tool are achievable.

## 2 Objectives

We aimed to describe how ROBINS-I is applied in a representative sample of systematic reviews of non-randomised studies and to investigate the extent to which ROBINS-I assessments can be mapped to specific results of the corresponding NRSIs in systematic review reports.

## 3 Methods

### 3.1 Criteria for including reviews

We included only reports of systematic reviews of non-randomised studies of intervention (NRSI) published during the second half of 2022 and reported in full text. To be regarded as a report of a systematic review, an article had to (i) have more than one author, (ii) include a clear statement of eligibility criteria for included studies, and (iii) provide an indication that the authors have sought to be comprehensive (either stated directly or inferred indirectly from the authors’ use of two or more bibliographic databases). NRSIs were defined as any studies comparing an intervention with a control or comparator intervention where allocation was not based on randomisation, such as cohort studies, quasi-randomised studies, case–control studies, or (controlled or uncontrolled) before–after studies. The reviews had to have reported the use of ROBINS-I for risk-of-bias assessment of the NRSI and have meta-analysis results that can be used in estimating the effect of the study intervention. We did not include non-English articles, systematic reviews that used only other critical appraisal tools, or systematic reviews that used ROBINS-I to examine studies that were not NRSIs. We also excluded any reviews that were unavailable or had missing appendices.

### 3.2 Review identification

In January 2023, we carried out a forward citation search in Web of Science for all articles that have cited the Sterne et al 2016 article describing ROBINS-I.^4^ The citation records were then exported to EndNote where we applied the eligibility criteria. We screened titles and abstracts of reviews published in the 6 months before the search date (July–December 2022).^9^ Screening was done by a single reviewer (ZIE or RJB), with all potentially excluded reviews being discussed by ZIE and RJB prior to exclusion.

### 3.3 Data extraction

We extracted data from the reviews, using a piloted data extraction form (Supplementary Material 1), on the following:

1. Review details: Study ID and authors’ contact, study designs included, type of intervention, outcomes assessed, and clinical area according to the ICD-10 categories.
2. Use of ROBINS-I: whether (i) there was evidence that a target trial was defined; (ii) key confounding factors for the review were pre-specified; (iii) all seven bias domains were assessed; and (iv) standard risk-of-bias judgements were used.
3. Reporting of ROBINS-I: whether (i) overall risk-of-bias judgements; (ii) domain-level judgements; (iii) justifications for judgements; and (iv) answers to signalling questions were reported, and whether these could be linked to specific results or outcomes of the corresponding NRSIs. ROBINS-I results were judged as being linked to specific results or outcomes if: ROBINS-I traffic lights were attached to forest plots, the caption of ROBINS-I results indicated the outcome of interest, or the review in question reported only one outcome.
4. Reviews were marked ‘yes’ or ‘no’ depending on whether or not they deviated from the ROBINS-I guidance. Those that provided insufficient information were marked ‘not reported’.

Initially, three reviewers (ZIE, JS, and JPTH) independently piloted the data extraction process on a sample of reviews to address any potential inconsistencies and ambiguities in data extraction. Agreement was good, and because the extracted data were straightforward (the purpose of the study is descriptive analysis and not establishing the effectiveness of treatment), it was deemed appropriate to continue data extraction by a single reviewer (ZIE or RJB). We did not examine the included NRSIs for missing information and did not contact review authors for clarifications.

During the peer review process, it was requested that we assess whether the implementation of ROBINS-I was carried out by one or more authors in each review as mandated by the Cochrane’s Methodological Expectations of Cochrane intervention reviews (MECIR)^10^ and what information on inter-rater agreement was reported. Therefore, we carried out a post hoc extraction of this information in 10% of the included reviews that were randomly selected.

### 3.4 Data analysis

We produced descriptive statistics on overall rates of adherence and deviations from ROBINS-I guidance and the reporting of its use in the reviews. Where possible, we assessed the ROBINS-I application according to study characteristics such as clinical area, intervention type, authors’ affiliation/location, and outcome type.

Our main outcome is the proportion of studies that deviated from the ROBINS-I guidance for the core tool. We regarded a review as adhering if it: assessed all seven bias domains (and no additional domains) and used the standard four levels of risk of bias (low, moderate, serious, and critical risk of bias). We also assessed the reporting of the use of ROBINS-I.

## 4 Results

**Figure 1:**
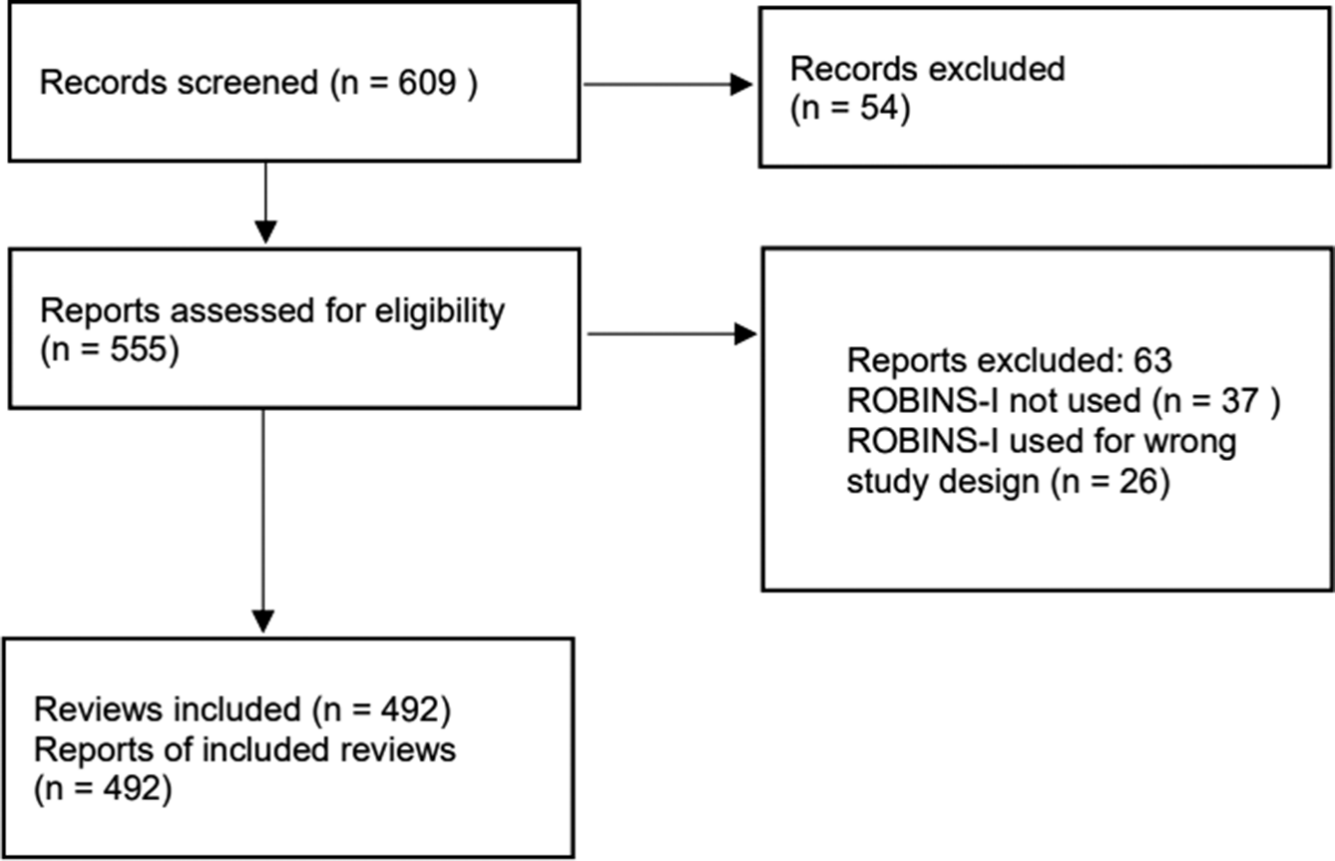
PRISMA study selection flowchart.

The citation search found 609 records that were published within the 6 months preceding the search date. We screened the records and found 555 publications meeting the eligibility criteria. We scrutinised full-text copies of these publications and excluded 63 (Supplementary Material 2). More than half of the excluded articles did not use ROBINS-I (59%), while the rest of the articles (41%) used ROBINS-I in systematic reviews that were not focused on assessing the effect of an intervention. We included 492 papers and carried out data extraction on these reviews. The study selection process is shown in the PRISMA flowchart (Figure 1).

The most common study design assessed with the ROBINS-I tool was cohort (*n* = 219 reviews). This was followed by controlled before and after (65 reviews), before and after (including interrupted time series) (63 reviews), case–control (49 reviews), and quasi-RCT (41 reviews). There were 242 reviews that included studies with other designs while 26 reviews did not state the design of the studies included. The reviews focused on a wide range of conditions and populations, including neoplasms (12%), diseases of the circulatory system (10%), diseases of the digestive system (10%) and factors influencing health status, and contact with health services (18%). Interventions studied were either pharmacological (26%), surgical (31%), psychosocial/behaviour change (7%), mixed (3%), or others (33%).

### 4.1 Deviation from ROBINS-I use

Among all the 492 included reviews, we found that 14 (3%) deviated from the seven standard domains, with 420 (85%) adhering to the domains and 58 (12%) providing insufficient information for us to judge (Table 1). We observed that 63 (13%) deviated from the ROBINS-I judgements compared with 403 (82%) adhering, and that seven (1%) deviated in other ways, due to partial use of either the signalling questions or the judgements.

**Table 1:** Deviations from ROBINS-I use (n = 492)

	Yes (%)	No (%)	Not reported (%)
Deviated from the seven standard domains (added, omitted, and/or modified)	14 (3)	420 (85)	58 (12)
Added additional domain(s)^a^	6 (1)	430 (87)	58 (12)
Omitted ROBINS-I domain(s)^a^	12 (2)	424 (86)	58 (12)
Modified ROBINS-I domain(s)^a^	6 (1)	428 (87)	58 (12)
Deviated from the risk-of-bias judgement categories	63 (13)	403 (82)	26 (5)
Deviated in any other way	7 (1)	444 (90)	41 (8)

a Some reviews deviated from the standard domains in more ways than one.

Of the 14 reviews that deviated from the standard ROBINS-I domains, six modified one or more ROBINS-I domains, 12 omitted domains, while six added non-ROBINS-I domains (Table 2). Omitted domains were either replaced with non-ROBINS-I domains or not replaced at all. There were also instances where domains were merged or split to create new domains.

**Table 2:** Deviation from ROBINS-I domains

			
Review	ROBINS-I domains							Domain modification	Additional domains
	D1	D2	D3	D4	D5	D6	D7		
Park 2022	🗶	🗶	🗶	✓	✓	✓	✓		
Saunders 2022	✓	✓	✓	🗶	✓	✓	✓		
Thielemann 2022	🗶	🗶	🗶	✓	✓	✓	✓		
Houttu 2022	✓	✓	✓	✓	✓	✓	🗶		
Wong 2022	✓	✓	✓	✓	✓	🗶	🗶		
Lin 2022	✓	✓	X	M	M	M	M	D4: ‘Bias due to knowledge of allocated interventions by participants and personnel’; D6: ‘Detection bias’; D5: ‘Attrition bias’; D7: ‘Reporting bias’	
Scuteri 2022	M	🗶	✓	✓	✓	✓	✓	Did not use D1 or D2 but used a domain ‘Baseline differences’	
Sousa 2022	✓	M	M	M	M	M	M	Described as a ‘simplified version’, D2/D5: ‘Selection bias’, D3/D6: ‘Information bias’ (relating to recall and detection bias separately), D7: ‘Reporting bias’.	
Li 2022	✓	✓	M	M	✓	✓	✓	Did not use D3 or D4 but added a domain ‘Bias from the interventions’; further details not provided.	
Psaltis 2022	✓	✓	✓	✓	✓	M	✓	D6: ‘Blinding of outcome assessment’	Added ‘Other bias’
Hensums 2022	🗶	✓	✓	✓	✓	✓	🗶		
Essibayi 2022	✓	✓	✓	🗶	✓	🗶	✓		Added ‘Correlation with clinical practice’
El-Andari 2022	M	M	🗶	🗶	M	M	🗶	D1: ‘Confounding variables’, D2: ‘Group selection’, D5: ‘Missing data’, D6: ‘Classification of outcomes’	Added domains: ‘Recruitment strategy’, ‘Inclusion and exclusion criteria’, ‘Sample size’, ‘Matching’, Statistical methods’, ‘Missing important outcomes’, ‘Follow-up time’ and ‘Incomplete follow-up’
Burns 2022	✓	M	✓	✓	✓	✓	✓	D2 was divided into two domains, ‘Sample selection’ and ‘Comparability of the groups’.	
Ostapenko 2022	✓	✓	✓	✓	✓	✓	✓		‘Other source of bias’ (rated as ‘n/a’ for every study)

*Note*: ‘X’: domain omitted; ✓: domain applied; ‘M’: domain modified.

Table 3 shows that the use of a ‘high’ (rather than a ‘serious’) risk-of-bias judgement alone or in combination with other non-standard judgement categories was a major reason for deviation in 55 (85%) out of the 64 reviews that deviated from the standard ROBINS-I judgement categories. The reviews that partially adhered to the ROBINS-I judgements (six reviews) and signalling questions (one review) were considered as having deviated in ‘other ways’. Full details on these reviews are outlined in Table 4.

**Table 3:** Deviation from ROBINS-I judgements

Judgement categories reported^a^	Reviews	References
Low/**Unclear or Uncertain/High**	18	Bin 2022, Burns 2022, Chen 2022a, Chen 2022b, Clark 2022, Essex 2022, Joyce 2022, Katoto 2022, Khajuria 2022, Lin 2022, Psaltis 2022, Ponugoti 2022, Poupore 2022a, Poupore 2022b, Poupore 2022c, Strawbridge 2022, Van Swol 2022, Yang 2022, Zhao 2022
Low/Moderate/**High**	15	Ali 2022, Augustus 2022, Cappannoli 2022, Condello 2022, Doty 2022, Garoufalia 2022, Herasevich 2022, Kaza 2022, Obaid 2022, Pugalendhi 2022, Saunders 2022, Shlobin 2022, Tonprasong 2022, van Weelden 2022, van de Heyning 2022
Low/Some concerns/**High**	5	Chang 2022a, Debeuf 2022, Oppici 2022, Sack 2022, Zhang 2022
Low/No information/**Some concerns/High**	2	Chang 2022b, Thielemann 2022^b^
Low/Moderate/**Unclear/High**	2	Bullock 2022, Taubin 2022
**Low/Probably low/Probably high/High**	1	Li 2022
Low/Moderate/**Some concerns/Unclear/High**	1	Huang 2022
Low/No information/**Some concerns**	1	de Oliveira 2022
Low/Moderate/No information/**High**	1	Khachatryan 2022
Low/No information/ **High**	1	Liu 2022
Low/No information/**Unclear/High**	1	Scuteri 2022
Moderate/Serious/**High**	1	Shieu 2022
Low/**Medium/High**	1	Byrd 2022
Low/**Intermediate/High**	1	Sprouse 2022
Low/Moderate/**Severe**	1	Fackler 2022
Low/**High**	1	Rehman 2022
**Not biased/Unclear/Biased**	1	Park 2022
**Yes/Probably yes/Probably no/No**	1	Sousa 2022
**Scores 1, 2, and 3**	1	Cleere 2022
**Justifications were used as judgements**	1	Idriss 2022
**Judgement categories were not clear**	6	Checinski 2022, Jeong 2022, Johnston 2022, (only **High** reported); El Andari 2022, Mattison 2022 (only Low/**Unclear or Unsure** reported); Lin 2022 (only Moderate/**High** reported)

a Non-ROBINS-I judgement categories are in bold;

b Thielman 2022: serious + critical were replaced with high.

**Table 4:** Deviation from ROBINS-I in other ways

Review	Judgements	Signalling questions
Koutoukidis 2022	Each domain of risk of bias was scored as 1, 2, 3, or 4 if they were judged as low, moderate, serious, or critical risk of bias respectively. Although the authors reported ROBINS-I judgements at the domain level, at the study level, the authors provided a total score, and the overall risk of bias was rated lower/higher than the median total score, for example, 11, higher (than the median score).	
Faulkner 2022	Each study was scored out of 11, with the final score incorporating study timeline (that is prospective/retrospective).	
Aiolfi 2022	Standard ROBINS-I judgements were used at study level; however, for the domains, ‘yes’, ‘probably yes’, ‘no’, ‘probably no’, and ‘no’ were used	
Aiolfi 2022b	Standard ROBINS-I judgements were used at study level, however, for the domains, ‘yes’, ‘probably yes’, ‘no’, ‘probably no’ and ‘no’ were used	
Ladenbauer 2022		Partial application of the signalling questions—It appears not all signalling questions in the ROBINS-I guidance were considered
Pitsios 2022	A total score was calculated for each study, according to the number of quality items fulfilled, divided by seven (number of bias domains), yielding a score between 0 and 1	
Qian, 2022	The authors applied a scoring system to the ROBINS-I judgement: The score of each domain ranged from 0 to 4, with ‘No information’ (0), ‘Low’ (1—low risk of bias), ‘Moderate’ (2—moderate risk of bias), ‘Serious’ (3—serious risk of bias), and ‘Critical’ (4—critical risk of bias)	

### 4.2 Reporting of ROBINS-I use

Only five reviews reported specification of a target trial (1%), and 99 listed important confounding factors to be considered in the domain of bias due to confounding (20%). Most of the reviews meeting our inclusion criteria reported ROBINS-I results (476; 97%), although 16 (3%) did not report any (Table 5). Of the reviews that reported ROBINS-I results, 88% did so by domain, and 89% used the standard ROBINS-I judgement categories. A smaller proportion provided justifications for risk-of-bias assessment (11%) and answers to individual signalling questions (3%).

**Table 5:** Reporting of ROBINS-I use and results

	Yes (%)	No (%)
*All reviews (n = 492)*		
Reported specification of a target trial	5 (1)	487 (99)
Stated the confounding factors of interest	99 (20)	393 (80)
Reported any ROBINS-I assessment results	476 (97)	16 (3)
*Reviews reporting ROBINS-I results (n = 476)*		
Reported ROBINS-I assessments by domain	418 (88)	58 (12)
Reported ROBINS-I judgements	424 (89)	52 (11)
Reported justifications for risk-of-bias assessment	52 (11)	424 (89)
Reported answers to signalling questions	16 (3)	460 (97)

### 4.3 Mapping of ROBINS-I assessments to systematic review results

Of the 476 reviews that reported ROBINS-I results, 371 (78%) linked these results to each individual study. However, in only 57 (12%) could we link ROBINS-I results to specific outcomes or results, and in only 36 (8%) of the reviews were we able to link ROBINS-I assessments to results contributing to meta-analyses. A further 203 (43%) of the reviews that presented ROBINS-I results by study alone carried out meta-analyses, while 73 (15%) of the reviews did not report a meta-analysis.

### 4.4 Post hoc analysis

We randomly selected 50 out of the 492 reviews and assessed whether there was information available on the number of authors involved in data extraction and inter-rater agreement. Of the 50 reviews selected information on the number of authors involved in data extraction was available in 35 (70%) reviews, and two or more authors were reported to have carried out data extraction in 34 of those reviews. None of the reviews reported on inter-rater agreement. The reviews rather described how disagreements between authors were resolved during the risk-of-bias assessment.

## 5 Discussion

This study provides a summary of the ways in which systematic review authors apply and report the use of the ROBINS-I tool. We found 492 systematic reviews meeting our inclusion criteria and assessed their application of the ROBINS-I guidance based on a set of criteria. We assessed the deviation of systematic reviews from ROBINS-I methods and the reporting of its use. Based on the criteria assessed, deviation from the standard ROBINS-I domains and judgements occurred in 3% and 14% of reviews respectively. Over 80% of the reviews reported ROBINS-I results, domain results, and ROBINS-I judgements. On the other hand, the specification of a target trial, stating of confounding factors, reporting of justifications of risk-of-bias assessment, and answers to signalling questions were only reported in 1%–20% of the reviews.

We were only able to link ROBINS-I assessments to specific study results in 8% of the reviews. This is of concern as the knowledge of the weaknesses of systematic review evidence is essential in decision-making. Risk-of-bias results that are outcome specific are more useful for decision-making unlike study-level risk-of-bias results, which tend to be more generic and not always context/outcome specific. We assessed ROBINS-I adherence using 10 criteria that were based on whether the reviews reported the methods in full, and only one out of the 492 included reviews fully adhered to all the criteria.^11^ One of the reasons for low adherence could be that ROBINS-I has been demonstrated to be difficult to apply.^12^ Some authors may not understand the importance of outcome-level application of the ROBINS-I tool, and others may opt for study-level ROBINS-I assessment for convenience especially when dealing with systematic reviews reporting multiple outcomes. Study level ROBINS-I assessment is unhelpful in the interpretation and implementation of systematic review evidence. This is because the effects of various sources of bias, such as confounders, attrition rate, and measurement of outcomes, could vary between outcomes. Failure to specify a target trial, state confounding factors, and report answers to signalling questions may have been due to authors’ reluctance to adopt these unique features, which distinguish ROBINS-I from older versions of similar domain-based tools. The implementation of these features may also have been underestimated due to the authors’ need to comply with strict journal guidelines on word limits and failure to consider other ways of making the information available, such as online supplements and data repositories.

We judged review authors’ adherence to the ROBINS-I guidance based on the level of reporting of the basic features of the tool. These criteria are merely basic requirements for ROBINS-I reporting alone. The use of the ROBINS-I tool requires more complex considerations. For example, users are required to consider whether co-interventions or the adjustment for confounders could result in a change in the estimated effect of the intervention, whether confounders have been measured, and the reliability of the measurement. The inability of authors to adhere to the basic criteria around reporting raises concerns about competence in dealing with the less obvious and more complex requirements of the application of ROBINS-I.

Our findings agree with results of Igelström et al.^8^ in ROBINS-I criteria with the greatest adherence. The study reported similar proportions to ours of systematic reviews that did not modify their risk-of-bias judgements and domains, in 124 reviews published in early 2020. Although our study covers additional aspects of the ROBINS-I guidance such as the specification of a target trial and reporting of answers to signalling questions, which were not considered by Igelström et al., an improvement in compliance with the ROBINS-I guidance is evident in our study compared with Igelström et al., with our study reporting at least twice the levels of adherence to domain specific RoB judgements, justification of judgements and reporting of confounding factors in Igelström et al. This could be due to the sample variation between the two studies given that our study included more recently published reviews. The adoption of ROBINS-I has probably improved with the passage of time as understanding and expertise has developed. Therefore, our study should be more representative of the way in which ROBINS-I methods and results are currently applied and reported by systematic review authors.

Five out of our 10 ROBINS-I criteria considered were adhered to by 20% or less of the included reviews. This poor compliance with the guidance raises concerns around whether the way the tool is currently applied in reviews fulfils its purpose of appraising the strengths and weaknesses of systematic reviews of NRSIs. Low levels of reporting of justification for different judgements as well as answers to signalling questions, further call into question the transparency of the critical appraisal process, which is a key feature of ROBINS-I. Whilst there appears to be a general improvement in compliance with the ROBINS-I guidance and reporting of results, evidence users may need to exercise caution in discerning fully compliant from non-compliant review evidence. More than half of the reviews reporting both ROBINS-I results and meta-analyses, some of which are linked to specific outcomes/results, provide data that can be used in carrying out risk of bias adjustments. This can pave the way for new knowledge on ROBINS-I and on the influence of specific types of bias on study results. A study on adherence to RoB 2 guidance^13^ shows that the problem of low compliance is not unique to the ROBINS-I tool alone and was found to improve with better instructions.^14^ Given that NRSIs are generally considered to have greater susceptibility to biases than randomised trials, it is just as important that they are assessed carefully for risk of bias if they are to be used to inform decision-making.

Strengths of our study include a large sample and meticulous data collection. However, there were several limitations to our study. We did not examine the quality of the ROBINS-I assessments by repeating them or examining the NRSIs on which they were based. However, there is evidence that inter-rater agreement in ROBINS-I application is moderate.^15^ Although we wrote a protocol for our own purposes, we did not register this and we did not specify *a priori* a definition for adherence. We also did not assess the aspect of the guidance related to co-intervention due to discussions around its potential exclusion from the forthcoming revision of the ROBINS-I tool. Although the included reviews appear to be diverse, it is unclear whether there is a time lag bias due to the sample being dominated by articles that were published during the latter half of the year. Initially, we did not address implementation issues such as the number of authors involved in ROBINS-I assessments as it is not part of the ROBINS-I guidance, therefore, not within the scope of our study. We acknowledge this as a limitation and have done a post-hoc analysis on a random sample of reviews to assess this.

## 6 Conclusion

Systematic review authors do not fully comply with the ROBINS-I guidance or report its use. The only review that met all the requirements of the ROBINS-I guidance was authored by a highly experienced team, including some of the developers of ROBINS-I. Clearer guidance for authors or simplification of the tool may improve compliance in future studies. Further training opportunities, such as online webinars, may help the situation in the future, although the extent to which users will seek such training in practice is unclear. It will be helpful to understand whether systematic reviews that do comply with all ROBINS-I features differ in the validity of their conclusions from those that achieve partial compliance. There is also a lack of understanding on whether the risk-of-bias judgements reported by authors are a true reflection of the limitations of the studies included in the reviews. The current application of ROBINS-I and the reporting of its use in systematic reviews of NRSIs creates concerns around how the evidence is being used in decision-making.

## Supporting information

Iheozor-Ejiofor et al. supplementary material 1 Iheozor-Ejiofor et al. supplementary material

Iheozor-Ejiofor et al. supplementary material 2 Iheozor-Ejiofor et al. supplementary material

## Data Availability

Data are available at https://doi.org/10.5281/zenodo.17077011.

## Supplementary material

The supplementary material for this article can be found at http://doi.org/10.1017/rsm.2025.10048.
