# Supplementary material for: The application of ROBINS-I guidance in systematic reviews of non-randomised studies: A descriptive study
Source: Res Synth Methods. 2025 Oct 22;17(2):265–76. doi: 10.1017/rsm.2025.10048 (PMC12873613; doi:10.1017/rsm.2025.10048)
Supplement: Iheozor-Ejiofor et al. supplementary material 1 — Iheozor-Ejiofor et al. supplementary material [file S1759287925100483sup001.docx]

# Data extraction form

| Review title |  |
| --- | --- |
| Study ID *(surname of first author and year first full report of study was published e.g. Smith 2001)* |  |
| Report ID |  |
| Report ID of other reports of this study including errata or retractions |  |
| Notes | |

#### **General Information**

| Date form completed *(mm/yyyy)* |  |
| --- | --- |
| Name/ID of person extracting data |  |
| Reference citation |  |
| Study authors contact details |  |
| Publication type *(e.g. full report, abstract, letter)* |  |
| Notes: | |

#### **Study eligibility**

| Study Characteristics | Eligibility criteria  *(Insert inclusion criteria for each characteristic as defined in the Protocol)* | Eligibility criteria met? | | | Notes |
| --- | --- | --- | --- | --- | --- |
|  |  | Yes | No | Unclear |  |
| Type of study | Systematic review on effect of intervention:   - more than one author - clear statement of eligibility criteria for included studies - indication that they have sought to be comprehensive (stated directly or inferred indirectly from the authors’ use of two or more databases).) |  |  |  |  |
|  | Non-randomised studies of interventions |  |  |  |  |
|  | Evidence of the use of ROBINS-I applied to included NRSI (in methods, results, or discussion) |  |  |  |  |
| INCLUDE  EXCLUDE  UNCLEAR | | | | | |
| Exclusion reasons collected specifically: | | | | | |
| - ROBINS-I used but for studies that are not NRSIs - ROBINS-I used but for NRSIs when interest is NOT in effect of the intervention | | | | | |

**DO NOT PROCEED IF STUDY EXCLUDED FROM REVIEW**

#### **ROBINS-I Assessment**

##### Methods

|  | Yes No | **Descriptions as stated in report/paper and l**ocation in text or source *(pg & ¶/fig/table/other)* |
| --- | --- | --- |
| **1 Did the authors report the use of the ROBINS-I tool?** |  |  |
| **METHODS** |  |  |
| **2.1 Did the authors specify a target trial?** |  |  |
| **2.2 Did the authors state the confounding factors of interest?** |  |  |
| **2.1 Did the authors use the seven standard domains? If no, state what domains were assessed.**  **Bias due to confounding Bias due to selection of participants into the studies   Bias due to classification of interventions    Bias due to deviation from intended intervention    Bias due to missing data   Bias due to measurement of outcome    Bias due to selection of the reported result** |  |  |
| **2.2 Did the authors use the standard risk of bias judgement categories (and names)? If no, state what categories were used.** |  |  |
| **2.3 Did the authors deviate from the standard tool in any other way? If yes, state how.**  **Low**  **Moderate**  **Serious**  **Critical**  **No information** |  |  |
| Results |  |  |
| **3.1 Did the authors report any ROBINS-I assessment results? If yes, state the location** |  |  |
| **3.2 Did the authors report ROBINS-I assessments by domain? If yes, state the location** |  |  |
| **3.3 Did the authors report ROBINS-I assessments by domain? If yes, state the domains assessed** |  |  |
| **3.4 Did the authors report ROBINS-I judgments?** |  |  |
| **3.5 Were justifications for risk of bias assessment reported?** |  |  |
| **3.6 Did the authors report answers to signalling questions?** |  |  |
| **3.7 To what can ROBINS-I assessments be linked?** | Study  Outcome (or subset of outcomes)  Specific result (estimate/CI)  None of these |  |
| **3.8 If ROBINS-I assessment can be linked to study results i.e outcome and/or specific result (above), is a meta-analysis presented (with results by study)?** |  |  |
| **3.9 If answer to 3.8 is yes, is it sufficiently reported for inclusion in a meta-epidemiological study?** |  |  |
| **Reported study design of included studies: Cohort**   **Case-control** **Before and After (including ITS)**  **Controlled Before and After**  **qRCT** **Other** **Not stated** | | |
| **Notes:** | | |

#### **PICO characteristics for the review**

##### Intervention Group 1

|  | Description as stated in report/paper | Category |
| --- | --- | --- |
| Population/Condition |  |  |
| Intervention |  |  |
| Clinical area |  |  |
| Notes: | | |
