# Supplementary material for: The application of ROBINS-I guidance in systematic reviews of non-randomised studies: A descriptive study
Source: Res Synth Methods. 2025 Oct 22;17(2):265–76. doi: 10.1017/rsm.2025.10048 (PMC12873613; doi:10.1017/rsm.2025.10048)
Supplement: Iheozor-Ejiofor et al. supplementary material 2 — Iheozor-Ejiofor et al. supplementary material [file S1759287925100483sup002.docx]

**LIST OF INCLUDED STUDIES**

1. Abdullatif V, Sur R, Abdullatif Z, Szabo S, Abbott J, Abdullatif VA, et al. The Safety and Efficacy of Endoscopic Combined Intrarenal Surgery (ECIRS) versus Percutaneous Nephrolithotomy (PCNL): A Systematic Review and Meta-Analysis. Adv Urol. 2022;2022.

2. Abu Arqub S, Gandhi V, Iverson M, Alam M, Allareddy V, Liu D, et al. Radiographic and histological assessment of root resorption associated with conventional and mini-screw assisted rapid palatal expansion: a systematic review. Eur J Orthodont. 2022;44(6):679-89.

3. Adamina M, Andreou A, Arezzo A, Christogiannis C, Di Lorenzo N, Gioumidou M, et al. EAES rapid guideline: systematic review, meta-analysis, GRADE assessment, and evidence-informed European recommendations on appendicitis in pregnancy. Surg Endosc. 2022;36(12):8699-712.

4. Adamyan L, Kasyan V, Pivazyan L, Isaeva S, Avetisyan J. Laser vaporization compared with other surgical techniques in women with ovarian endometrioma: a systematic review and meta-analysis. Arch Gynecol Obstet. 2023;308(2):413-25.

5. Adesuyan M, Jani Y, Alsugeir D, Cheung E, Chui C, Howard R, et al. Antihypertensive Agents and Incident Alzheimer's Disease: A Systematic Review and Meta-Analysis of Observational Studies. JPAD-J Prev Alzheimers Dis. 2022;9(4):715-24.

6. Adler A, Bhatia V, Chandrakantan A, Nathanson B, Ouellette L, Austin P, et al. Association of Analgesic Block With the Incidence of Complications Following Hypospadias Surgery; A Meta-Analysis. Urology. 2022;166:11-7.

7. Ahmed F, McMillan T, Guenther B, Dearborn P, Ahmed FS, McMillan TM, et al. Cognitive Performance following Single- or Multi-Session Exercise Intervention in Middle Age: A Systematic Review. Exp Aging Res. 2024;50(1):28-64.

8. Ahmed K, Ibad H, Suchal Z, Gosain A, Ahmed KS, Ibad H, et al. Implementation of 3D Printing and Computer-Aided Design and Manufacturing (CAD/CAM) in Craniofacial Reconstruction. J Craniofac Surg. 2022;33(6):1714-9.

9. Ahn N, Nolde M, Krause E, Guntner F, Gunter A, Tauscher M, et al. Do proton pump inhibitors increase the risk of dementia? A systematic review, meta-analysis and bias analysis. Br J Clin Pharmacol. 2023;89(2):602-16.

10. Aijtink V, Rutten V, Meijer B, de Jong R, Isaac J, Polak W, et al. Safety of Intraoperative Blood Salvage During Liver Transplantation in Patients With Hepatocellular Carcinoma A Systematic Review and Meta-analysis. Ann Surg. 2022;276(2):239-45.

11. Aiolfi A, Sozzi A, Bonitta G, Lombardo F, Cavalli M, Cirri S, et al. Linear- versus circular-stapled esophagogastric anastomosis during esophagectomy: systematic review and meta-analysis. Langenbecks Arch Surg. 2022;407(8):3297-309.

12. Aiolfi A, Sozzi A, Lombardo F, Lanzaro A, Panizzo V, Bonitta G, et al. Laparoscopic paraesophageal hernia repair with absorbable mesh: a systematic review. Video-Assist Thorac Surg. 2022;7.

13. Ali O, Gupta S, Brain K, Lifford K, Paranjothy S, Dolwani S, et al. Acceptability of alternative technologies compared with faecal immunochemical test and/or colonoscopy in colorectal cancer screening: A systematic review. J Med Screen. 2023;30(1):14-27.

14. Aljamali H, Eslick G, Weltman M, Aljamali H, Eslick GD, Weltman M. Meta-analysis: hepatitis B reactivation in patients receiving biological therapy. Aliment Pharmacol Ther. 2022;56(7):1104-18.

15. Alqahtani K, Shaheen E, Morgan N, Shujaat S, Politis C, Jacobs R, et al. Impact of orthognathic surgery on root resorption: A systematic review. J Stomatol Oral Maxillofac Surg. 2022;123(5):e260-e7.

16. Amani B, Amani B, Amani B, Amani B. Efficacy and safety of sotrovimab in patients with COVID-19: A rapid review and meta-analysis. Rev Med Virol. 2022;32(6).

17. Amaratunga H, Bostock K, Cunich M, Steffens D, Carey S, Amaratunga H, et al. Systematic review of service improvements for home enteral tube feeding in adults. Nutr Clin Pract. 2023;38(2):329-39.

18. Anand P, Kaushal M, Ramaswamy V, Pullattayil S, Razak A, Trevisanuto D, et al. Nasal Cannula with Long and Narrow Tubing for Non-Invasive Respiratory Support in Preterm Neonates: A Systematic Review and Meta-Analysis. Children-Basel. 2022;9(10).

19. Anitua E, Allende M, Eguia A, Alkhraisat M, Anitua E, Allende M, et al. Bone-Regenerative Ability of Platelet-Rich Plasma Following Sinus Augmentation with Anorganic Bovine Bone: A Systematic Review with Meta-Analysis. Bioengineering-Basel. 2022;9(10).

20. Archer V, Cloutier Z, Berg A, McKechnie T, Wiercioch W, Eskicioglu C, et al. Short-stay compared to long-stay admissions for loop ileostomy reversals: a systematic review and meta-analysis. Int J Colorectal Dis. 2022;37(10):2113-24.

21. Archontakis-Barakakis P, Li W, Kalaitzoglou D, Tzelves L, Manolopoulos A, Giannopoulos S, et al. Effectiveness and safety of intracranial events associated with the use of direct oral anticoagulants for atrial fibrillation: A systematic review and meta-analysis of 92 studies. Br J Clin Pharmacol. 2022;88(11):4663-75.

22. Arias-Vazquez P, Tovilla-Zarate C, Castillo-Avila R, Legorreta-Ramirez B, Lopez-Narvaez M, Arcila-Novelo R, et al. Hypertonic Dextrose Prolotherapy, an Alternative to Intra-Articular Injections With Hyaluronic Acid in the Treatment of Knee Osteoarthritis Systematic Review and Meta-analysis. Am J Phys Med Rehabil. 2022;101(9):816-25.

23. Arrese I, Garcia-Garcia S, Cepeda S, Sarabia R, Arrese I, Garcia-Garcia S, et al. Treatment of unruptured middle cerebral artery aneurysms: Systematic review in an attempt to perform a network meta-analysis. Front Surg. 2022;9.

24. Asmat K, Dhamani K, Gul R, Froelicher E, Asmat K, Dhamani K, et al. The effectiveness of patient-centered care vs. usual care in type 2 diabetes self-management: A systematic review and meta-analysis. Front Public Health. 2022;10.

25. Athanasiou A, Veroniki A, Efthimiou O, Kalliala I, Naci H, Bowden S, et al. Comparative effectiveness and risk of preterm birth of local treatments for cervical intraepithelial neoplasia and stage IA1 cervical cancer: a systematic review and network meta-analysis. Lancet Oncol. 2022;23(8):1097-108.

26. Augustus E, Haynes E, Guell C, Morrissey K, Murphy M, Halliday C, et al. The Impact of Nutrition-Based Interventions on Nutritional Status and Metabolic Health in Small Island Developing States: A Systematic Review and Narrative Synthesis. Nutrients. 2022;14(17).

27. Bäcke P, Bruschettini M, Blomqvist YT, Olsson E. Interventions for the management of Pain and Sedation in Newborns undergoing Therapeutic hypothermia for hypoxic-ischemic encephalopathy (IPSNUT): protocol of a systematic review. Syst Rev. 2022;11(1):101.

28. Bao X, Sun F, Yao H, Wang D, Liu H, Tang G, et al. Distal end of Double-J ureteral stent position on ureteral stent-related symptoms: A systematic review and meta-analysis. Front Surg. 2022;9.

29. Barello S, Anderson G, Acampora M, Bosio C, Guida E, Irace V, et al. The effect of psychosocial interventions on depression, anxiety, and quality of life in hemodialysis patients: a systematic review and a meta-analysis. Int Urol Nephrol. 2023;55(4):897-912.

30. Basurrah AA, Di Blasi Z, Lambert L, Murphy M, Warren MA, Setti A, et al. The effects of positive psychology interventions in Arab countries: A systematic review. Applied Psychology: Health and Well-Being. 2023;15(2):803-21.

31. Baums M, Aquilina J, Perez-Prieto D, Sleiman O, Geropoulos G, Totlis T, et al. Risk analysis of periprosthetic knee joint infection (PJI) in total knee arthroplasty after preoperative corticosteroid injection: a systematic review A study performed by the Early-Osteoarthritis group of ESSKA-European Knee Associates section. Arch Orthop Trauma Surg. 2023;143(5):2683-91.

32. Bekteshi S, Konings M, Karlsson P, Van Criekinge T, Dan B, Monbaliu E, et al. Teleintervention for users of augmentative and alternative communication devices: A systematic review. Dev Med Child Neurol. 2023;65(2):171-84.

33. Belcher B, Kang D, Yunker A, Dieli-Conwright C, Belcher BR, Kang D-W, et al. Interventions to Reduce Sedentary Behavior in Cancer Patients and Survivors: a Systematic Review. Curr Oncol Rep. 2022;24(11):1593-605.

34. Bestetti A, de Moura D, Proenca I, Junior E, Ribeiro I, Sasso J, et al. Endoscopic Resection Versus Surgery in the Treatment of Early Gastric Cancer: A Systematic Review and Meta-Analysis. Front Oncol. 2022;12.

35. Bezerra P, Vieira T, dos Santos F, Ribeiro I, de Sousa S, Valenca A, et al. The impact of oral health education on the incidence and severity of oral mucositis in pediatric cancer patients: a systematic review and meta-analysis. Support Care Cancer. 2022;30(11):8819-29.

36. Biebuyck G, Neradova A, de Fijter C, Jakulj L, Biebuyck GKM, Neradova A, et al. Impact of telehealth interventions added to peritoneal dialysis-care: a systematic review. BMC Nephrol. 2022;23(1).

37. Bilgin C, Hardy N, Hutchison K, Pederson J, Mebane A, Olaniran P, et al. First-line thrombectomy strategy for distal and medium vessel occlusions: a systematic review. J NeuroInterventional Surg. 2023;15(6):539-46.

38. Bin L, Fei J, Zhao L, Hong R, Yang W, Bin L, et al. Comparative effectiveness and safety of open triple-branched stent graft technique with stented elephant trunk implantation in treating Stanford type A aortic dissection: A trial sequential meta-analysis. J Card Surg. 2022;37(12):5210-7.

39. Boeykens M, Keller E, Bosio A, Wiseman O, Contreras P, Ventimiglia E, et al. Impact of Ureteral Stent Material on Stent-related Symptoms. Eur Urol Open Sci. 2022;45:108-17.

40. Bollen E, Awad L, Langridge B, Butler P, Bollen E, Awad L, et al. The intraoperative use of augmented and mixed reality technology to improve surgical outcomes: A systematic review. Int J Med Robot Comput Assist Surg. 2022;18(6).

41. Bosco F, Giustra F, Via R, Lavia A, Capella M, Sabatini L, et al. Could anterior closed-wedge high tibial osteotomy be a viable option in patients with high posterior tibial slope who undergo anterior cruciate ligament reconstruction? A systematic review and meta-analysis. Eur J Orthop Surg Traumatol. 2023;33(6):2201-14.

42. Boutari C, Pappas P, Anastasilakis D, Mantzoros C, Boutari C, Pappas PD, et al. Statins' efficacy in non-alcoholic fatty liver disease: A systematic review and meta-analysis. Clin Nutr. 2022;41(10):2195-206.

43. Bruno A, Benson A, Metz T, Blue N, Bruno AM, Benson AE, et al. Adjunct Therapy at Time of Examination-Indicated Cervical Cerclage in Singleton Pregnancies: A Systematic Review and Meta-analysis. Am J Perinatol. 2022;39(16):1719-25.

44. Buckley B, Lane D, Calvert P, Zhang J, Gent D, Mullins C, et al. Effectiveness and Safety of Apixaban in over 3.9 Million People with Atrial Fibrillation: A Systematic Review and Meta-Analysis. J Clin Med. 2022;11(13).

45. Budi D, Rofananda I, Pratama N, Sutanto H, Hariftyani A, Desita S, et al. Ozone as an adjuvant therapy for COVID-19: A systematic review and meta-analysis. Int Immunopharmacol. 2022;110.

46. Buffone F, Monacis D, Tarantino A, Dal Farra F, Bergna A, Agosti M, et al. Osteopathic Treatment for Gastrointestinal Disorders in Term and Preterm Infants: A Systematic Review and Meta-Analysis. Healthcare. 2022;10(8).

47. Bugan B, Cekirdekci E, Onar L, Barcin C, Bugan B, Cekirdekci EI, et al. Transcatheter Tricuspid Valve Replacement for Tricuspid Regurgitation: A Systematic Review and Meta-analysis. Anat J Cardiol. 2022;26(7):505-+.

48. Bullock G, Sell T, Zarega R, Reiter C, King V, Wrona H, et al. Kinesiophobia, Knee Self-Efficacy, and Fear Avoidance Beliefs in People with ACL Injury: A Systematic Review and Meta-Analysis. Sports Med. 2022;52(12):3001-19.

49. Burns E, Feeley C, Hall P, Vanderlaan J, Burns E, Feeley C, et al. Systematic review and meta-analysis to examine intrapartum interventions, and maternal and neonatal outcomes following immersion in water during labour and waterbirth. BMJ Open. 2022;12(7).

50. Byrd K, Shieh J, Mork S, Pincus L, O'Meara L, Atkins M, et al. Fish and Fish-Based Products for Nutrition and Health in the First 1000 Days: A Systematic Review of the Evidence from Low and Middle-Income Countries. Adv Nutr. 2022;13(6):2458-87.

51. Caccavella V, Giordano M, Colicchio G, Izzo A, D'Ercole M, Rapisarda A, et al. Palliative Surgery for Drug-Resistant Epilepsy in Adult Patients. A Systematic Review of the Literature and a Pooled Analysis of Outcomes. World Neurosurg. 2022;163:132-+.

52. Caiado V, Santos A, Moreira-Marconi E, Moura-Fernandes M, Seixas A, Taiar R, et al. Effects of Physical Exercises Alone on the Functional Capacity of Individuals with Obesity and Knee Osteoarthritis: A Systematic Review. Biology-Basel. 2022;11(10).

53. Caminiti C, Maglietta G, Diodati F, Puntoni M, Marcomini B, Lazzarelli S, et al. The Effects of Patient-Reported Outcome Screening on the Survival of People with Cancer: A Systematic Review and Meta-Analysis. Cancers. 2022;14(21).

54. Candeloro M, Valeriani E, Monreal M, Ageno W, Riva N, Lopez-Reyes R, et al. Anticoagulant therapy for splanchnic vein thrombosis: an individual patient data meta-analysis. Blood Adv. 2022;6(15):4516-23.

55. Cappannoli L, Galli M, Zito A, Restivo A, Princi G, Laborante R, et al. Venoarterial extracorporeal membrane oxygenation (VA-ECMO) with vs. without left ventricular unloading by Impella: a systematic review and meta-analysis. Eur Heart J-Qual Care Clin Outcomes. 2023;9(4):358-66.

56. Caruso G, Palaia I, Bogani G, Tomao F, Perniola G, Panici P, et al. Systematic lymph node dissection during interval debulking surgery for advanced epithelial ovarian cancer: a systematic review and meta-analysis. J Gynecol Oncol. 2022;33(5).

57. Castelli L, Galasso L, Mule A, Ciorciari A, Fornasini F, Montaruli A, et al. Sleep and spa therapies: What is the role of balneotherapy associated with exercise? A systematic review. Front Physiol. 2022;13.

58. Chahal B, Aydin A, Amin M, Ong K, Khan A, Khan M, et al. Transfer of open and laparoscopic skills to robotic surgery: a systematic review. J Robot Surg. 2023;17(4):1207-25.

59. Chammas P, Hadouiri N, Chammas M, -Pascual S, Stirling P, Nover L, et al. Proximal row carpectomy generates better mid- to long-term outcomes than four-corner arthrodesis for post-traumatic wrist arthritis: A meta-analysis. Orthop Traumatol-Surg Res. 2022;108(7).

60. Chan K, Shlobin N, Dahdaleh N, Chan KS, Shlobin NA, Dahdaleh NS. Diagnosis and management of isolated C1 fractures: A systematic review. J Craniovertebral Junction Spine. 2022;13(3):233-44.

61. Chang E, Ali R, Seibert J, Berkman N, Chang E, Ali R, et al. Interventions to Improve Outcomes for High-Need, High-Cost Patients: A Systematic Review and Meta-Analysis. J Gen Intern Med. 2023;38(1):185-94.

62. Chang E, Rains C, Ali R, Wines R, Kahwati L, Chang E, et al. Minimally invasive sacroiliac joint fusion for chronic sacroiliac joint pain: a systematic review. Spine Journal. 2022;22(8):1240-53.

63. Chang J, Fu S, Li X, Li S, Wang X, Zhou Z, et al. The differential effects of sleep deprivation on pain perception in individuals with or without chronic pain: A systematic review and meta-analysis. Sleep Med Rev. 2022;66.

64. Checa C, Canelo-Aybar C, Suclupe S, Ginesta-Lopez D, Berenguera A, Castells X, et al. Effectiveness and Cost-Effectiveness of Case Management in Advanced Heart Failure Patients Attended in Primary Care: A Systematic Review and Meta-Analysis. Int J Environ Res Public Health. 2022;19(21).

65. Checinski M, Checinska K, Turosz N, Kaminska M, Nowak Z, Sikora M, et al. Autologous Stem Cells Transplants in the Treatment of Temporomandibular Joints Disorders: A Systematic Review and Meta-Analysis of Clinical Trials. Cells. 2022;11(17).

66. Chen K, Miake-Lye I, Begashaw M, Zimmerman F, Larkin J, McGrath E, et al. Association of Promoting Housing Affordability and Stability With Improved Health Outcomes A Systematic Review. JAMA Netw Open. 2022;5(11).

67. Chen L, Chen Y, Hu S, Lin M, Lee P, Chiang A, et al. In search of a better CPAP interface: A network meta-analysis comparing nasal masks, nasal pillows and oronasal masks. J Sleep Res. 2022;31(6).

68. Chen M, Zhang M, Chen H, Chen M, Zhang M, Chen H. EFFICIENCY OF LASER PHOTOCOAGULATION ON THE PREVENTION OF RETINAL DETACHMENT IN ACUTE RETINAL NECROSIS A Systematic Review and Meta-Analysis. Retin-J Retin Vitr Dis. 2022;42(9):1702-8.

69. Chen S, Guo L, Zhong L, Shen L, Zeng Q, Lai L, et al. Preparation of small bowel capsule endoscopy (SBCE) with simethicone: A meta-analysis. Clin Res Hepatol Gastroenterol. 2022;46(10).

70. Chen X, Xiang H, Yang M, Chen X, Xiang H, Yang M. Topical cetirizine for treating androgenetic alopecia: A systematic review. J Cosmet Dermatol. 2022;21(11):5519-26.

71. Cheng H, French C, Salam A, Dawson S, McAleenan A, McGuinness L, et al. Lack of Evidence for Ribavirin Treatment of Lassa Fever in Systematic Review of Published and Unpublished Studies. Emerg Infect Dis. 2022;28(8):1559-68.

72. Chesdachai S, Go J, Hassett L, Baddour L, DeSimone D, Chesdachai S, et al. The utility of postoperative systemic antibiotic prophylaxis following cardiovascular implantable electronic device implantation: A systematic review and meta-analysis. PACE-Pacing Clin Electrophysiol. 2022;45(8):940-9.

73. Chierici A, Frontali A, Granieri S, Facciorusso A, Angelis N, Cotsoglou C, et al. Postoperative morbidity and mortality after pancreatoduodenectomy with pancreatic duct occlusion compared to pancreatic anastomosis: a systematic review and meta-analysis. Hpb. 2022;24(9):1395-404.

74. Chilkoti G, Mohta M, Ahmad Z, Saxena A, Chilkoti GT, Mohta M, et al. Awake Prone-Positioning in Patients on Non-Invasive Ventilation for Management of SARS-CoV-2 Pneumonia: A Systematic Review. Adv Respir Med. 2022;90(4):362-75.

75. Choo C, Chen Y, McHoney M, Choo CSC, Chen Y, McHoney M. Delayed versus early repair of inguinal hernia in preterm infants: A systematic review and meta-analysis. J Pediatr Surg. 2022;57(11):527-33.

76. Choong E, Shu X, Leung K, Lo E, Choong EKM, Shu X, et al. Oral health-related quality of life (OHRQoL) after rehabilitation with removable partial dentures (RPDs): A systematic review and meta-analysis. J Dent. 2022;127.

77. Chung S, Candelaria D, Gallagher R, Chung S, Candelaria D, Gallagher R. Women's Health-Related Quality of Life Substantially Improves With Tailored Cardiac Rehabilitation A SYSTEMATIC REVIEW AND META-ANALYSIS. J Cardiopulm Rehabil Prev. 2022;42(4):217-26.

78. Clapp B, Portela R, Sharma I, Nakanishi H, Marrero K, Schauer P, et al. Risk of non-hormonal cancer after bariatric surgery: meta-analysis of retrospective observational studies. Br J Surg. 2022;110(1):24-33.

79. Clark I, Brougham M, Spears N, Mitchell R, Clark I, Brougham MFH, et al. The impact of vincristine on testicular development and function in childhood cancer. Hum Reprod Update. 2023;29(2):233-45.

80. Claus B, Stahlschmidt L, Dunford E, Major J, Harbeck-Weber C, Bhandari R, et al. Intensive interdisciplinary pain treatment for children and adolescents with chronic noncancer pain: a preregistered systematic review and individual patient data meta-analysis. Pain. 2022;163(12):2281-301.

81. Cleere E, Davey M, Young O, Lowery A, Kerin M, Cleere EF, et al. Intra-operative nerve monitoring and recurrent laryngeal nerve injury during thyroid surgery: a network meta-analysis of prospective studies. Langenbecks Arch Surg. 2022;407(8):3209-19.

82. Condello F, Cacia M, Sturla M, Terzi R, Sanz-Sanchez J, Reimers B, et al. Simultaneous Radial and Ipsilateral Ulnar Artery Compression versus Isolated Radial Artery Compression after Conventional Radial Access for Coronary Angiography and/or Intervention: A Systematic Review and Meta-Analysis. J Clin Med. 2022;11(23).

83. Constantinescu D, Costello J, Dalling A, Wagner J, Al-Hardan W, Carvajal J, et al. The efficacy of patient specific instrumentation (PSI) in total hip arthroplasty (THA): A systematic review and meta-analysis. J Orthop. 2022;34:404-13.

84. Constantinescu D, Pavlis W, Rizzo M, Vanden Berge D, Barnhill S, Hernandez V, et al. The role of commercially available smartphone apps and wearable devices in monitoring patients after total knee arthroplasty: a systematic review. EFORT Open Rev. 2022;7(7):481-90.

85. Cooper M, Avery L, Scott J, Ashley K, Jordan C, Errington L, et al. Effectiveness and active ingredients of social prescribing interventions targeting mental health: a systematic review. BMJ Open. 2022;12(7).

86. Cristina T, Mara T, Arianna S, Gennaro S, Rosaria C, Pantaleo G, et al. Impact of waterbirth on post-partum hemorrhage, genital trauma, retained placenta and shoulder dystocia: A systematic review and meta-analysis. Eur J Obstet Gynecol Reprod Biol. 2022;276:26-37.

87. Crookes D, Stanhope K, Suglia S, Crookes DM, Stanhope KK, Suglia SF. Immigrant-Related Policies and the Health Outcomes of Latinx Adults in the United States: A Systematic Review. Epidemiology. 2022;33(4):593-605.

88. Csader S, Korhonen S, Kaarniranta K, Schwab U, Csader S, Korhonen S, et al. The Effect of Dietary Supplementations on Delaying the Progression of Age-Related Macular Degeneration: A Systematic Review and Meta-Analysis. Nutrients. 2022;14(20).

89. Cuello C, De Jesus O, Espinosa A, Thomas R, Murray G, Pastrana E, et al. Prognosis and Outcome of Cervical Primary Extraosseous Intradural Extramedullary Ewing Sarcoma: A Systematic Review. Cureus J Med Sci. 2022;14(7).

90. D'Amico F, Barucco G, Licheri M, Valsecchi G, Zaraca L, Mucchetti M, et al. Opioid Free Anesthesia in Thoracic Surgery: A Systematic Review and Meta Analysis. J Clin Med. 2022;11(23).

91. da Silva Higa KT, Böhme FAF, Paschoa S, Conte ACR, Santos VB, Avelar AFM. Dark nighttime interventions and sleep quality in intensive care unit patients: A systematic review and meta‐analysis. Nurs Crit Care. 2022.

92. Dall'Ora C, Saville C, Rubbo B, Turner L, Jones J, Griffiths P, et al. Nurse staffing levels and patient outcomes: A systematic review of longitudinal studies. Int J Nurs Stud. 2022;134.

93. Darriba I, Seidel A, Moreno F, Botelho J, Machado V, Mendes J, et al. Influence of low insertion torque values on survival rate of immediately loaded dental implants: A systematic review and meta-analysis. J Clin Periodontol. 2023;50(2):158-69.

94. Datrino L, Orlandini M, Andrade M, Santos C, Modesto V, Tavares G, et al. Two- versus three-field lymphadenectomy for esophageal cancer. A systematic review and meta-analysis of early and late results. J Surg Oncol. 2022;126(1):76-89.

95. Davies PSE, Pennington R, Dhadwal AS, Chokotho L, Nyamulani N, Mpanga C, et al. Clinical outcomes of ankle fractures in sub-Saharan Africa: a systematic review. Eur J Orthop Surg Traumatol. 2023;33(3):547-57.

96. de Bock E, Herman E, Bastian O, Filipe M, Vriens M, Richir M, et al. Systematic review and meta-analysis determining the effect of implemented COVID-19 guidelines on surgical oncology volumes and clinical outcomes. Surg Oncol-Oxf. 2022;45.

97. de Bruin R, van Dalen S, Franx S, Ramaswamy V, Simons S, Flint R, et al. The Risk for Neonatal Hypoglycemia and Bradycardia after Beta-Blocker Use during Pregnancy or Lactation: A Systematic Review and Meta-Analysis. Int J Environ Res Public Health. 2022;19(15).

98. de Leon B, del Pino-Sedeno T, Serrano-Perez P, Alvarez C, Bejarano-Quisoboni D, Trujillo-Martin M, et al. Effectiveness of interventions to improve medication adherence in adults with depressive disorders: a meta-analysis. BMC Psychiatry. 2022;22(1).

99. de Oliveira A, Arcanjo F, Rodrigues M, Silva A, Hall P, Queiroz de Oliveira AF, et al. Use of autologous platelet-rich plasma in androgenetic alopecia in women: a systematic review and meta-analysis. J Dermatol Treat. 2023;34(1):2138692.

100. de Oliveira L, Calixto-Lima L, Cunha G, da Silva N, De Souza-Silva R, Fonseca T, et al. Effects of specialised nutritional interventions in patients with incurable cancer: a systematic review. BMJ Support Palliat Care. 2022;12(4):388-402.

101. de Souza R, Correa B, Melo P, Pousa P, de Mendonca T, Rodrigues L, et al. The treatment of atypical hemolytic uremic syndrome with eculizumab in pediatric patients: a systematic review. Pediatr Nephrol. 2023;38(1):61-75.

102. Debeuf R, Swinnen E, Plattiau T, De Smedt A, De Waele E, Roggeman S, et al. EFFECT OF PHYSICAL THERAPY ON IMPAIRMENTS IN COVID-19 PATIENTS FROM INTENSIVE CARE TO HOME REHABILITATION: A RAPID REVIEW. J Rehabil Med. 2022;54.

103. Del Rey Y, Parize H, Pedrazzi V, dos Reis A, do Nascimento C, Del Rey YC, et al. Clinical and In Situ Oral Biofilm Formation on Dental Implant Abutment Materials: A Systematic Review. Int J Oral Maxillofac Implants. 2022;37(4):639-52.

104. den Hartog F, Sneiders D, Darwish E, Yurtkap Y, Menon A, Muysoms F, et al. Favorable Outcomes After Retro-Rectus (Rives-Stoppa) Mesh Repair as Treatment for Noncomplex Ventral Abdominal Wall Hernia, a Systematic Review and Meta-analysis. Ann Surg. 2022;276(1):55-65.

105. Derk G, Barton A, An R, Fang H, Ashrafi S, Wilund K, et al. The Safety and Efficacy of Clonidine in Hemodialysis Patients: A Systematic Review and Meta-Analysis. Pharmacology. 2022;107(11):545-55.

106. Dhillon J, Kraeutler M, Belk J, Scillia A, Dhillon J, Kraeutler MJ, et al. Umbilical Cord-Derived Stem Cells for the Treatment of Knee Osteoarthritis: A Systematic Review. Orthop J Sports Med. 2022;10(7).

107. Di Chiaro B, Santiago G, Santiago C, Zelko I, Choudhary A, Purnell C, et al. A Systematic Review of Primary Rhinoplasty in Patients With Bilateral Cleft Lip. J Craniofac Surg. 2022;33(8):2406-10.

108. Di Spirito F, Caggiano M, Di Palo M, Contaldo M, D'Ambrosio F, Martina S, et al. Oral Lesions in Pediatric Subjects: SARS-CoV-2 Infection and COVID-19 Vaccination. Appl Sci-Basel. 2022;12(18).

109. Doerr F, Stange S, Michel M, Schlachtenberger G, Menghesha H, Wahlers T, et al. Redefining the role of surgery in early small-cell lung cancer. Langenbecks Arch Surg. 2022;407(7):2663-71.

110. Domenicucci R, Ferrandes F, Sarlo M, Borella E, Belacchi C, Domenicucci R, et al. Efficacy of ICT-based interventions in improving psychological outcomes among older adults with MCI and dementia: A systematic review and meta-analysis. Ageing Res Rev. 2022;82.

111. Domnich A, de Waure C, Domnich A, de Waure C. Comparative effectiveness of adjuvanted versus high-dose seasonal influenza vaccines for older adults: a systematic review and meta-analysis. Int J Infect Dis. 2022;122:855-63.

112. Doppen M, Kung S, Maijers I, John M, Dunphy H, Townsley H, et al. Cannabis in Palliative Care: A Systematic Review of Current Evidence. J Pain Symptom Manage. 2022;64(5):E260-E84.

113. dos Santos C, dos Santos L, Tavares G, Tristao L, Orlandini M, Serafim M, et al. Prophylactic thoracic duct obliteration and resection during esophagectomy: What is the impact on perioperative risks and long-term survival? A systematic review and meta-analysis. J Surg Oncol. 2022;126(1):90-8.

114. Doty B, Bass J, Ryan T, Zhang A, Wilcox H, Doty B, et al. Systematic review of suicide prevention studies with data on youth and young adults living in low-income and middle-income countries. BMJ Open. 2022;12(9).

115. Dudas C, Czumbel L, Kiss S, Gede N, Hegyi P, Martha K, et al. Clinical bracket failure rates between different bonding techniques: a systematic review and meta-analysis. Eur J Orthodont. 2023;45(2):175-85.

116. Dunworth K, Fimbres D, Trotta R, Hollins A, Shammas R, Allori A, et al. Systematic Review and Critical Appraisal of the Evidence Base for Nasoalveolar Molding (NAM). Cleft Palate-Craniofac J. 2024;61(4):654-77.

117. Duong Q, Pittet L, Curtis N, Zimmermann P, Duong QA, Pittet LF, et al. Antibiotic exposure and adverse long-term health outcomes in children: A systematic review and meta-analysis. J Infect. 2022;85(3):213-300.

118. Ebeid A, Cole E, Stallwood-Hall C, Ebeid A, Cole E, Stallwood-Hall C. The efficacy of weight-based enoxaparin dosing for venous thromboembolism prophylaxis in trauma patients: A systematic review and meta-analysis. J Trauma Acute Care Surg. 2022;93(2):E71-E9.

119. El Sammak S, Mualem W, Michalopoulos G, Romero J, Ha C, Hunt C, et al. Rescue therapy with novel waveform spinal cord stimulation for patients with failed back surgery syndrome refractory to conventional stimulation: a systematic review and meta-analysis. J Neurosurg-Spine. 2022;37(5):670-9.

120. EL-Andari R, Bozso S, Fialka N, Kang J, MacArthur R, Meyer S, et al. Coronary Artery Revascularization in Heart Transplant Patients: A Systematic Review and Meta-Analysis. Cardiology. 2022;147(3):348-63.

121. Elfil M, Ghaith H, Elsayed H, Aladawi M, Elmashad A, Patel N, et al. Intravenous thrombolysis plus mechanical thrombectomy versus mechanical thrombectomy alone for acute ischemic stroke: A systematic review and updated meta-analysis of clinical trials. Interv Neuroradiol. 2024;30(4):550-63.

122. Ellard O, Dennison C, Tuomainen H, Ellard OB, Dennison C, Tuomainen H. Review: Interventions addressing loneliness amongst university students: a systematic review. Child Adolesc Ment Health. 2023;28(4):512-23.

123. Ellis E, Frye T, Ellis EE, Frye TP. Role of multi-parametric magnetic resonance imaging fusion biopsy in active surveillance of prostate cancer: a systematic review. Ther Adv Urol. 2022;14.

124. Elmholt S, Nielsen T, Lind M, Elmholt SB, Nielsen TG, Lind M. Fixed-loop vs. adjustable-loop cortical button devices for femoral fixation in ACL reconstruction - a systematic review and meta-analysis. J Exp Orthop. 2022;9(1).

125. Emonena H, Ojo O, Emonena H, Ojo O. The Efficacy of Tele-Monitoring in Maintaining Glycated Haemoglobin Levels in Patients with Type 2 Diabetes Mellitus: A Systematic Review. Int J Environ Res Public Health. 2022;19(24).

126. Ercolino O, Baccin E, Alfier F, Villani P, Trevisanuto D, Cavallin F, et al. Thermal servo-controlled systems in the management of VLBW infants at birth: A systematic review. Front Pediatr. 2022;10.

127. Eslami Jahromi M, Ayatollahi H. Impact of telecare interventions on quality of life in older adults: a systematic review. Aging Clin Exp Res. 2023;35(1):9-21.

128. Essex R, Weldon S, Thompson T, Kalocsanyiova E, McCrone P, Deb S, et al. The impact of health care strikes on patient mortality: A systematic review and meta-analysis of observational studies. Health Serv Res. 2022;57(6):1218-34.

129. Essibayi M, Lanzino G, Keser Z, Essibayi MA, Lanzino G, Keser Z. Vitamin K antagonist versus novel oral anticoagulants for management of cervical artery dissection: Interactive systematic review and meta-analysis. Eur Stroke J. 2022;7(4):349-57.

130. Fackler N, Chin G, Karasavvidis T, Bohlen H, Smith E, Amirhekmat A, et al. Outcomes of Arthroscopic Lysis of Adhesions for the Treatment of Postoperative Knee Arthrofibrosis: A Systematic Review. Orthop J Sports Med. 2022;10(9).

131. Fawke J, Wyllie J, Udaeta E, Rudiger M, Ersdal H, Wright M, et al. Suctioning of clear amniotic fluid at birth: A systematic review. Resusc Plus. 2022;12.

132. Fernandez J, Molina-Martin A, Rocha-de-Lossada C, Rodriguez-Vallejo M, Pinero D, Fernandez J, et al. Clinical outcomes of presbyopia correction with the latest techniques of presbyLASIK: a systematic review. Eye. 2023;37(4):587-96.

133. Filges T, Dalgaard N, Viinholt B, Filges T, Dalgaard NT, Viinholt BCA. Outreach programs to improve life circumstances and prevent further adverse developmental trajectories of at-risk youth in OECD countries: A systematic review. Campbell Syst Rev. 2022;18(4).

134. Fiore M, Sambri A, Filippini M, Morante L, Giannini C, Paolucci A, et al. Are Static Spacers Superior to Articulated Spacers in the Staged Treatment of Infected Primary Knee Arthroplasty? A Systematic Review and Meta-Analysis. J Clin Med. 2022;11(16).

135. Fitzpatrick A, Cooper C, Vasilunas N, Ritchie B, Fitzpatrick A, Cooper C, et al. Describing the Impact of Maternal Hyperimmune Globulin and Valacyclovir on the Outcomes of Cytomegalovirus Infection in Pregnancy: A Systematic Review( ). Clin Infect Dis. 2022;75(8):1467-80.

136. Flierman I, Gieteling E, Van Rijn M, Van Grootven B, van Doorne I, Jamaludin F, et al. Effectiveness of transmural team-based palliative care in prevention of hospitalizations in patients at the end of life: A systematic review and meta-analysis. Palliat Med. 2023;37(1):75-87.

137. Flowers D, McCallister E, Christopherson R, Ware E, Flowers DW, McCallister E, et al. The Safety and Effectiveness of Early, Progressive Weight Bearing and Implant Choice after Traumatic Lower Extremity Fracture: A Systematic Review. Bioengineering-Basel. 2022;9(12).

138. Flynn J, Larach J, Kong J, Waters P, McCormick J, Warrier S, et al. Patient-Related Functional Outcomes After Robotic-Assisted Rectal Surgery Compared With a Laparoscopic Approach: A Systematic Review and Meta-analysis. Dis Colon Rectum. 2022;65(10):1191-204.

139. Frassini S, Calabretto F, Granieri S, Fugazzola P, Vigano J, Fazzini N, et al. Intraperitoneal chemotherapy in the management of pancreatic adenocarcinoma: A systematic review and meta-analysis. Ejso. 2022;48(9):1911-21.

140. Frey M, Ahsan M, Bergeron H, Lin J, Li X, Fowlkes R, et al. Cascade Testing for Hereditary Cancer Syndromes: Should We Move Toward Direct Relative Contact? A Systematic Review and Meta-Analysis. J Clin Oncol. 2022;40(35):4129-+.

141. Frodl A, Siegel M, Fuchs A, Wagner F, Schmal H, Izadpanah K, et al. Minced Cartilage Is a One-Step Cartilage Repair Procedure for Small Defects in the Knee-A Systematic-Review and Meta-Analysis. J Pers Med. 2022;12(11).

142. Frost K, Hamilton R, Hughes S, Jamieson C, Rafferty P, Troise O, et al. Systematic review of high-dose amikacin regimens for the treatment of Gram-negative infections based on EUCAST dosing recommendations. Eur J Hosp Pharm. 2023;30(4):189-95.

143. Frountzas M, Liatsou E, Schizas D, Pergialiotis V, Vailas M, Kritikos N, et al. The impact of surgery delay on survival of resectable pancreatic cancer: A systematic review of observational studies. Surg Oncol-Oxf. 2022;45.

144. Fusini F, Girardo M, Aprato A, Masse A, Lorenzi A, Messina D, et al. Percutaneous Cement Discoplasty in Degenerative Spinal Disease: Systematic Review of Indications, Clinical Outcomes, and Complications. World Neurosurg. 2022;168:219-26.

145. Gadot R, Najera R, Hirani S, Anand A, Storch E, Goodman W, et al. Efficacy of deep brain stimulation for treatment-resistant obsessive-compulsive disorder: systematic review and meta-analysis. J Neurol Neurosurg Psychiatry. 2022;93(11):1166-73.

146. Galjart B, Hoppener D, Aerts J, Bangma C, Verhoef C, Grunhagen D, et al. Follow-up strategy and survival for five common cancers: A meta-analysis. Eur J Cancer. 2022;174:185-99.

147. Galvain T, Hill R, Donegan S, Lisboa P, Lip G, Czanner G, et al. Efficacy and Safety of Anticoagulants in Patients with Atrial Fibrillation and History of Falls or Risk of Falls: A Systematic Review and Multilevel Meta-Analysis. Drug Saf. 2022;45(11):1349-62.

148. Ganga A, Leary O, Sastry R, Asaad W, Svokos K, Oyelese A, et al. Antibiotic prophylaxis in penetrating traumatic brain injury: analysis of a single-center series and systematic review of the literature. Acta Neurochir. 2023;165(2):303-13.

149. Garel N, McAnulty C, Greenway K, Lesperance P, Miron J, Rej S, et al. Efficacy of ketamine intervention to decrease alcohol use, cravings, and withdrawal symptoms in adults with problematic alcohol use or alcohol use disorder: A systematic review and comprehensive analysis of mechanism of actions. Drug Alcohol Depend. 2022;239.

150. Garoufalia Z, Gefen R, Emile S, Silva-Alvarenga E, Freund M, Horesh N, et al. Outcomes of graciloplasty in the treatment of fecal incontinence: a systematic review and meta-analysis of the literature. Tech Coloproctology. 2023;27(6):429-41.

151. Garoufalia Z, Gefen R, Emile S, Silva-Alvarenga E, Horesh N, Freund M, et al. Gracilis muscle interposition for complex perineal fistulas: A systematic review and meta-analysis of the literature. Colorectal Dis. 2023;25(4):549-61.

152. Gatti M, Fornaro G, Viale P, Pea F, Giannella M, Gatti M, et al. Clinical efficacy of renal dosing adjustments of ceftazidime-avibactam in patients affected by carbapenem-resistant Gram-negative infections: A systematic review and meta-analysis of observational studies. Br J Clin Pharmacol. 2023;89(2):617-29.

153. Gauhar V, Pirola G, Scarcella S, De Angelis M, Giulioni C, Rubilotta E, et al. Nephrostomy tube versus double J ureteral stent in patients with malignant ureteric obstruction. A systematic review and meta-analysis of comparative studies. Int Braz J Urol. 2022;48(6):903-14.

154. Gauhar V, Teoh J, Mulawkar P, Tak G, Wroclawski M, Robles-Torres J, et al. Comparison and outcomes of dusting versus stone fragmentation and extraction in retrograde intrarenal surgery: results of a systematic review and meta-analysis. Cent Eur J Urol. 2022;75(3):317-27.

155. Ge C, Du K, Luo M, Shen K, Zhou Y, Guo K, et al. Serologic response and safety of COVID-19 vaccination in HSCT or CAR T-cell recipients: a systematic review and meta-analysis. Exp Hematol Oncol. 2022;11(1).

156. Gefen R, Garoufalia Z, Zhou P, Watson K, Emile S, Wexner S, et al. Treatment of enterocutaneous fistula: a systematic review and meta-analysis. Tech Coloproctology. 2022;26(11):863-74.

157. George S, Wilson M, Humphreys S, Gibbons K, Long E, Schibler A, et al. Apnoeic oxygenation during paediatric intubation: A systematic review. Front Pediatr. 2022;10.

158. Geri T, Botticchio A, Rossettini G, Pournajaf S, Pellicciari L, Di Antonio S, et al. Pressure Pain Threshold of the Upper Trapezius Trigger Point: A Systematic Review with Meta-Analysis of Baseline Values and Their Modification after Physical Therapy. J Clin Med. 2022;11(23).

159. Geropoulos G, Esagian S, Skarentzos K, Ziogas I, Katsaros I, Kosmidis D, et al. Video-assisted thoracoscopic versus open sleeve lobectomy for non-small cell lung cancer: A systematic review and meta-analysis from six comparative studies. Asian Cardiovasc Thorac Ann. 2022;30(8):881-93.

160. Ghaith H, Gabra M, Ebada M, Dada O, Al-Shami H, Bahbah E, et al. Tranexamic acid for patients with aneurysmal subarachnoid hemorrhage: a systematic review and meta-analysis of 2991 patients. Int J Neurosci. 2024;134(7):763-76.

161. Glenner-Frandsen A, With C, Gunnarsson T, Hostrup M, Glenner-Frandsen A, With C, et al. The Effect of Monophasic Oral Contraceptives on Muscle Strength and Markers of Recovery After Exercise-Induced Muscle Damage: A Systematic Review. Sports Health. 2023;15(3):318-27.

162. Goel K, Pek V, Shlobin N, Chen J, Wang A, Ibrahim G, et al. Clinical utility of intraoperative electrocorticography for epilepsy surgery: A systematic review and meta-analysis. Epilepsia. 2023;64(2):253-65.

163. Grange L, Chapelle C, Ollier E, Zufferey P, Douillet D, Killian M, et al. Adjusted versus fixed doses of LMWHs in trauma patients: A systematic review and meta-analysis. Anaesth Crit Care Pain Med. 2022;41(6).

164. Gravestock P, Moore L, Harding C, Veeratterapillay R, Gravestock P, Moore L, et al. Xanthogranulomatous pyelonephritis: a review and meta-analysis with a focus on management. Int Urol Nephrol. 2022;54(10):2445-56.

165. Gu J, Liu H, Lu H, Gu J, Liu H, Lu H. Can Even a Small Amount of Greenery Be Helpful in Reducing Stress? A Systematic Review. Int J Environ Res Public Health. 2022;19(16).

166. Gumiero J, de Oliveira B, Neto P, Pandini R, Gerbasi L, Figueiredo M, et al. Timing of resection of synchronous colorectal liver metastasis: A systematic review and meta-analysis. J Surg Oncol. 2022;126(1):175-88.

167. Guo D, Fan L, Le Q, Huang C, Guo D-F, Fan L-W, et al. Transjugular intrahepatic portosystemic shunt for the prevention of rebleeding in patients with cirrhosis and portal vein thrombosis: Systematic review and meta-analysis. Front Pharmacol. 2022;13.

168. Guo L, Chen S, Zhong L, He K, Li Y, Chen W, et al. Vitamin D intake as well as circulating 25-hydroxyvitamin D level and risk for the incidence and recurrence of colorectal cancer precursors: A meta-analysis. Front Med. 2022;9.

169. Haghbin H, Zakirkhodjaev N, Husain F, Lee-Smith W, Aziz M, Haghbin H, et al. Risk of Gastrointestinal Bleeding with Concurrent Use of NSAID and SSRI: A Systematic Review and Network Meta-Analysis. Dig Dis Sci. 2023;68(5):1975-82.

170. Hahn J, Jo Y, Yoo S, Shin J, Yu Y, Ah Y, et al. Risk of major adverse events associated with gabapentinoid and opioid combination therapy: A systematic review and meta-analysis. Front Pharmacol. 2022;13.

171. Haig S, Hallett N, Haig S, Hallett N. Use of sensory rooms in adult psychiatric inpatient settings: A systematic review and narrative synthesis. Int J Ment Health Nurs. 2023;32(1):54-75.

172. Hammer S, Toussaint M, Vollsaeter M, Tvedt M, Roksund O, Reychler G, et al. EXERCISE TRAINING IN DUCHENNE MUSCULAR DYSTROPHY: A SYSTEMATIC REVIEW AND META-ANALYSIS. J Rehabil Med. 2022;54.

173. Hauk D, Groschner A, Hauk D, Groschner A. How effective is learner-controlled instruction under classroom conditions? A systematic review. Learn Motiv. 2022;80.

174. Hawes C, Gomes A, Byham-Gray L, Henderson S, Hawes C, Gomes A, et al. The effect of oral nutrition supplements and appetite stimulants on weight status among pediatric cancer patients: a systematic review. Nutr Clin Pract. 2023;38(4):761-74.

175. He Z, Qiu S, Huang Z, Zhang G, An Q, Qu F, et al. Comparison between chronic spontaneous urticaria and chronic induced urticaria on the efficacy of omalizumab treatment: A systematic review and meta-analysis. Dermatol Ther. 2022;35(12):e15928.

176. Hensums M, de Mooij B, Kuijper S, Fekkes M, Overbeek G, Hensums M, et al. What Works for Whom in School-Based Anti-bullying Interventions? An Individual Participant Data Meta-analysis. Prev Sci. 2023;24(8):1435-46.

177. Herasevich S, Lipatov K, Pinevich Y, Lindroth H, Tekin A, Herasevich V, et al. The Impact of Health Information Technology for Early Detection of Patient Deterioration on Mortality and Length of Stay in the Hospital Acute Care Setting: Systematic Review and Meta-Analysis*. Crit Care Med. 2022;50(8):1198-209.

178. Heyne S, Macherey S, Meertens M, Braumann S, Niessen F, Tichelbacker T, et al. Coronary angiography after cardiac arrest without ST-elevation myocardial infarction: a network meta-analysis. Eur Heart J. 2023;44(12):1040-54.

179. Hidayat K, Zhou Y, Du H, Qin L, Shi B, Li Z, et al. A systematic review and meta-analysis of observational studies of the association between the use of incretin-based therapies and the risk of pancreatic cancer. Pharmacoepidemiol Drug Saf. 2023;32(2):107-25.

180. Hobbs C, Armitage J, Hood B, Jelbert S, Hobbs C, Armitage J, et al. A systematic review of the effect of university positive psychology courses on student psychological wellbeing. Front Psychol. 2022;13.

181. Honorio F, Tustumi F, Pinheiro J, Marques S, Glina F, Henriques A, et al. Esophagojejunostomy after total gastrectomy: A systematic review and meta-analysis comparing hand-sewn and stapled anastomosis. J Surg Oncol. 2022;126(1):161-7.

182. Houttu V, Bouts J, Vali Y, Daams J, Grefhorst A, Nieuwdorp M, et al. Does aerobic exercise reduce NASH and liver fibrosis in patients with non-alcoholic fatty liver disease? A systematic literature review and meta-analysis. Front Endocrinol. 2022;13.

183. Huang C, Chen H, Yang Y, Huang C, Chen I, Yang Y. Doripenem in the Treatment of Patients with Nosocomial Pneumonia: A Meta-Analysis. J Clin Med. 2022;11(14).

184. Huang H, Zhao N, Li Q, Qiao Q, Zhang J, Guo C, et al. The Therapeutic Effectiveness Using Fluorescence-Guided Surgery for MRONJ. Biomed Res Int. 2022;2022.

185. Huang S, Chen L, Tseng C, Chen C, Yuan L, Shau W, et al. Risk of cognitive impairment in men with advanced prostate cancer treated with NHAs: A systematic review and network meta-analysis. CTS-Clin Transl Sci. 2023;16(2):313-25.

186. Humphrey M, MacDonald G, Neville H, Helwig M, Ramsey T, MacKinnon H, et al. A Systematic Review of Antimicrobial Stewardship Interventions to Improve Management of Bacteriuria in Hospitalized Adults. Ann Pharmacother. 2023;57(7):855-66.

187. Huynh K, Nategh L, Jamadar S, Stout J, Georgiou-Karistianis N, Lampit A, et al. Cognition-oriented treatments and physical exercise on cognitive function in Huntington's disease: a systematic review. J Neurol. 2023;270(4):1857-79.

188. Hwang B, Williams M, Tian D, Yan T, Misfeld M, Hwang B, et al. Coronary artery bypass surgery for acute coronary syndrome: A network meta-analysis of on-pump cardioplegic arrest, off-pump, and on-pump beating heart strategies. J Card Surg. 2022;37(12):5290-9.

189. Idriss S, Reynard P, Marx M, Mainguy A, Joly C, Ionescu E, et al. Short- and Long-Term Effect of Cochlear Implantation on Disabling Tinnitus in Single-Sided Deafness Patients: A Systematic Review. J Clin Med. 2022;11(19).

190. Illés K, Meznerics FA, Dembrovszky F, Fehérvári P, Bánvölgyi A, Csupor D, et al. Mastoid Obliteration Decreases the Recurrent and Residual Disease: Systematic Review and Meta-analysis. Laryngoscope. 2023;133(6):1297-305.

191. Iness A, Abaricia J, Sawadogo W, Iness C, Duesberg M, Cyrus J, et al. The Effect of Hospital Visitor Policies on Patients, Their Visitors, and Health Care Providers During the COVID-19 Pandemic: A Systematic Review. Am J Med. 2022;135(10):1158-+.

192. Ismayl G, Kim W, Iqbal M, Sajid S, Ismayl G, Kim WJ, et al. Early Versus Delayed Treatment for Gartland Type III Supracondylar Humeral Fractures in Children: A Systematic Review and Meta-analysis. Indian J Orthop. 2022;56(11):1871-81.

193. Jamialahmadi T, Abbasifard M, Reiner Z, Rizzo M, Eid A, Sahebkar A, et al. The Effects of Statin Treatment on Serum Ferritin Levels: A Systematic Review and Meta-Analysis. J Clin Med. 2022;11(17).

194. Jau P, Chang S, Jau P-Y, Chang S-C. The effectiveness of acupuncture point stimulation for the prevention of postoperative sore throat A meta-analysis. Medicine (Baltimore). 2022;101(28).

195. Jawad M, Alnefeesi Y, Ceban F, Lui L, Jaberi S, Di Vincenzo J, et al. Lumateperone for the Treatment of Adults With Schizophrenia: a Systematic Review. Curr Psychiatry Rep. 2022;24(8):359-68.

196. Jensen L, Maier A, Lomstein A, Graillon T, Hrachova M, Bota D, et al. Somatostatin analogues in treatment-refractory meningioma: a systematic review with meta-analysis of individual patient data. Neurosurg Rev. 2022;45(5):3067-81.

197. Jiin Jeong E-YYLPB-HK, Yae-Na H. The Effects of a Tailored Activity Program for Dementia: A Systematic Review and Meta-Analysis. Physical \& Occupational Therapy In Geriatrics. 2023;41(2):280--91.

198. Johnston H, Takefala T, Kelly J, Keating S, Coombes J, Macdonald G, et al. The Effect of Diet and Exercise Interventions on Body Composition in Liver Cirrhosis: A Systematic Review. Nutrients. 2022;14(16).

199. Johnstone S, Dela Cruz G, Kalb N, Tyagi S, Potenza M, George T, et al. A systematic review of gender-responsive and integrated substance use disorder treatment programs for women with co-occurring disorders. Am J Drug Alcohol Abuse. 2023;49(1):21-42.

200. Jones D, Pinkham M, Wallen M, Hart N, Joseph R, Strodl E, et al. Benefits of supportive strategies for carers of people with high-grade glioma: a systematic review Strategies for addressing the needs of high-grade glioma carers. Support Care Cancer. 2022;30(12):10359-78.

201. Jones O, Hoyle P, Jones OP, Hoyle PJ. Azithromycin as an adjunct to subgingival professional mechanical plaque removal in the treatment of grade C periodontitis: a systematic review and meta-analysis. J Periodontal Implant Sci. 2022;52(5):352-69.

202. Joyce D, De Freitas S, Woo E, Tang T, Tubassam M, Walsh S, et al. Ultrasound-guided foam sclerotherapy as a therapeutic modality in venous ulceration. Surg J R Coll Surg Edinb Irel. 2022;20(5):E206-E13.

203. Jung J, McCutcheon K, Borofsky M, Young S, Golzarian J, Kim M, et al. Prostatic arterial embolisation for men with benign prostatic hyperplasia: a Cochrane review. BJU Int. 2023;131(1):32-45.

204. Kaiser L, Conrad S, Neugebauer E, Pietsch B, Pieper D, Kaiser L, et al. Interprofessional collaboration and patient-reported outcomes in inpatient care: a systematic review. Syst Rev. 2022;11(1).

205. Kang L, Shang W, Gao P, Wang Y, Liu J, Liu M, et al. Immunogenicity and Safety of COVID-19 Vaccines among People Living with HIV: A Systematic Review and Meta-Analysis. Vaccines. 2022;10(9).

206. Kao D, Protzuk O, O'Connell R, Kao DS, Protzuk OA, O'Connell RS. Clinical outcomes of cemented vs. uncemented reverse total shoulder arthroplasty for proximal humerus fractures: a systematic review. Eur J Orthop Surg Traumatol. 2023;33(5):2063-8.

207. Kara O, Gursen C, Cetin S, Tascioglu E, Muftuoglu S, Damiano D, et al. The effects of power exercises on body structure and function, activity and participation in children with cerebral palsy: an ICF-based systematic review. Disabil Rehabil. 2023;45(22):3705-18.

208. Karaszewska D, Cleintuar P, Oudijn M, Lok A, van Elburg A, Denys D, et al. Efficacy and safety of deep brain stimulation for treatment-refractory anorexia nervosa: a systematic review and meta-analysis. Transl Psychiatr. 2022;12(1).

209. Karpodini C, Dinas P, Angelopoulou E, Wyon M, Haas A, Bougiesi M, et al. Rhythmic cueing, dance, resistance training, and Parkinson's disease: A systematic review and meta-analysis. Front Neurol. 2022;13.

210. Katoto P, Brand A, Byamungu L, Tamuzi J, Mahwire T, Kitenge M, et al. Safety of COVID-19 Pfizer-BioNtech (BNT162b2) mRNA vaccination in adolescents aged 12-17 years: A systematic review and meta-analysis. Human Vaccines Immunother. 2022;18(6):2144039.

211. Kaza N, Htun V, Miyazawa A, Simader F, Porter B, Howard J, et al. Upgrading right ventricular pacemakers to biventricular pacing or conduction system pacing: a systematic review and meta-analysis. Europace. 2023;25(3):1077-86.

212. Kermansaravi M, Valizadeh R, Farazmand B, Mousavimaleki A, Taherzadeh M, Wiggins T, et al. De Novo Inflammatory Bowel Disease Following Bariatric Surgery: a Systematic Review and Meta-analysis. Obes Surg. 2022;32(10):3426-34.

213. Khachatryan V, Muazzam A, Hamal C, Velugoti L, Tabowei G, Gaddipati G, et al. The Role of Regorafenib in the Management of Advanced Gastrointestinal Stromal Tumors: A Systematic Review. Cureus J Med Sci. 2022;14(9).

214. Khaddad A, Bernhard J, Margue G, Michiels C, Ricard S, Chandelon K, et al. A survey of augmented reality methods to guide minimally invasive partial nephrectomy. World J Urol. 2023;41(2):335-43.

215. Khajuria A, Krzak A, Reddy R, Lai K, Wignakumar T, Rohrich R, et al. Piezoelectric Osteotomy versus Conventional Osteotomy in Rhinoplasty: A Systematic Review and Meta-analysis. Prs-Glob Open. 2022;10(11).

216. Khojaly R, Rowan F, Hassan M, Hanna S, Mac Niocail R, Khojaly R, et al. Weight-bearing Allowed Following Internal Fixation of Ankle Fractures, a Systematic Literature Review and Meta-Analysis. Foot Ankle Int. 2022;43(9):1143-56.

217. Khormali M, Heidari S, Ahmadi S, Bafrani M, Baigi V, Sharif-Alhoseini M, et al. N-methyl-D-aspartate receptor antagonists in improving cognitive deficits following traumatic brain injury: a systematic review. Brain Inj. 2022;36(9):1071-88.

218. Kim C, Yoon L, Michels K, Tranfield W, Jacobs J, May F, et al. The Impact of Prebiotic, Probiotic, and Synbiotic Supplements and Yogurt Consumption on the Risk of Colorectal Neoplasia among Adults: A Systematic Review. Nutrients. 2022;14(22).

219. Kim E, Park S, Kim S, Choi Y, Cho J, Kim G, et al. Is altitude a determinant of the health benefits of nature exposure? A systematic review and meta-analysis. Front Public Health. 2022;10.

220. Kim H, Venkatesulu B, McMillan M, Verma V, Lin S, Chang J, et al. Local Therapy for Oligoprogressive Disease: A Systematic Review of Prospective Trials. Int J Radiat Oncol Biol Phys. 2022;114(4):676-83.

221. Kim J, Lee M, Gao B, Yadav P, Ming J, Rickard M, et al. Comparison of continuous and interrupted suture techniques in pyeloplasty: a systematic review and meta-analysis. Pediatr Surg Int. 2022;38(9):1209-15.

222. Kim K, Belley-Cote E, Gupta S, Pandey A, Alsagheir A, Makhdoum A, et al. Mechanical versus bioprosthetic valves in chronic dialysis: a systematic review and meta-analysis. Can J Surg. 2022;65(4):E450-E9.

223. Kim R, An V, Petchell J, Kim RG, An VVG, Petchell JF. Fibular fixation in mid and distal extra-articular tibia fractures-A systematic review and meta-analysis. Foot Ankle Surg. 2022;28(7):809-16.

224. Kochman M, Kasprzak M, Kielar A, Kochman M, Kasprzak M, Kielar A. ACL Reconstruction: Which Additional Physiotherapy Interventions Improve Early-Stage Rehabilitation? A Systematic Review. Int J Environ Res Public Health. 2022;19(23).

225. Kok B, Wolthuis D, Bosch F, van der Hoeven H, Blans M, Kok B, et al. POCUS in dyspnea, nontraumatic hypotension, and shock; a systematic review of existing evidence. Eur J Intern Med. 2022;106:9-38.

226. Kolahdouzan K, Chavoshi M, Bayani R, Darzikolaee N, Kolahdouzan K, Chavoshi M, et al. Low-Dose Whole Lung Irradiation for Treatment of COVID-19 Pneumonia: A Systematic Review and Meta-Analysis. Int J Radiat Oncol Biol Phys. 2022;113(5):946-59.

227. Kong Y, Lv T, Li M, Zhao L, Meng T, Wu S, et al. Systematic review and meta-analysis: impact of anti-viral therapy on portal hypertensive complications in HBV patients with advanced chronic liver disease. Hepatol Int. 2022;16(5):1052-63.

228. Kouskoura T, Ochsner T, Verna C, Pandis N, Kanavakis G, Kouskoura T, et al. The effect of orthodontic treatment on facial attractiveness: a systematic review and meta-analysis. Eur J Orthodont. 2022;44(6):636-49.

229. Koutoukidis D, Jebb S, Zimmerman M, Otunla A, Henry J, Ferrey A, et al. The association of weight loss with changes in the gut microbiota diversity, composition, and intestinal permeability: a systematic review and meta-analysis. Gut Microbes. 2022;14(1).

230. Kovacs N, Nemeth D, Foldi M, Nagy B, Bunduc S, Hegyi P, et al. Selective intraoperative cholangiography should be considered over routine intraoperative cholangiography during cholecystectomy: a systematic review and meta-analysis. Surg Endosc. 2022;36(10):7126-39.

231. Kulper-Schiek W, Piechotta V, Pilic A, Batke M, Dreveton L, Geurts B, et al. Facing the Omicron variant-how well do vaccines protect against mild and severe COVID-19? Third interim analysis of a living systematic review. Front Immunol. 2022;13.

232. Kum A, De Moura D, Proenca I, Aikawa M, Sanchez-Luna S, Ribeiro I, et al. Gastroesophageal Reflux Waning Over Time in Endoscopic Versus Surgical Myotomy for the Treatment of Achalasia: A Systematic Review and Meta-Analysis. Cureus J Med Sci. 2022;14(11).

233. Kuramatsu J, Gerner S, Ziai W, Bardutzky J, Sembill J, Sprugel M, et al. Association of Intraventricular Fibrinolysis With Clinical Outcomes in Intracerebral Hemorrhage: An Individual Participant Data Meta-Analysis. Stroke. 2022;53(9):2876-86.

234. Kusi G, Atenafu E, Mensah A, Lee C, Viswabandya A, Puts M, et al. The effectiveness of psychoeducational interventions on caregiver-oriented outcomes in caregivers of adult cancer patients: A systematic review and meta-analysis. Psycho-Oncol. 2023;32(2):189-202.

235. Kuzminskaite E, Gathier A, Cuijpers P, Penninx B, Ammerman R, Brakemeier E, et al. Treatment efficacy and effectiveness in adults with major depressive disorder and childhood trauma history: a systematic review and meta-analysis. Lancet Psychiatry. 2022;9(11):860-73.

236. Labadie J, Ibrahim S, Worley B, Kang B, Rakita U, Rigali S, et al. Evidence-Based Clinical Practice Guidelines for Laser-Assisted Drug Delivery. JAMA Dermatol. 2022;158(10):1193-201.

237. Ladenbauer S, Singer J, Ladenbauer S, Singer J. Can Mindfulness-Based Stress Reduction Influence the Quality of Life, Anxiety, and Depression of Women Diagnosed with Breast Cancer? -A Review. Curr Oncol. 2022;29(10):7779-93.

238. Lam P, Zhou N, Yiu C, Wong H, Lam PPY, Zhou N, et al. Impact of Antiretroviral Therapy on Oral Health among Children Living with HIV: A Systematic Review and Meta-Analysis. Int J Environ Res Public Health. 2022;19(19).

239. Lan T, Tsou P, Tam K, Huang T, Lan T-C, Tsou P-H, et al. Effect of Urea Cream on Hand-Foot Syndrome in Patients Receiving Chemotherapy A Meta-analysis. Cancer Nurs. 2022;45(5):378-86.

240. Lang X, Qiao B, Ge Z, Yan J, Zhang Y, Lang X, et al. Clinical Effects of Photofunctionalization on Implant Stability and Marginal Bone Loss: Systematic Review and Meta-Analysis. J Clin Med. 2022;11(23).

241. Leal J, Farkas B, Mastikhina L, Flanagan J, Skidmore B, Salmon C, et al. Risk of transmission of respiratory viruses during aerosol-generating medical procedures (AGMPs) revisited in the COVID-19 pandemic: a systematic review. Antimicrob Resist Infect Control. 2022;11(1).

242. Lee H, Win T, Balakrishnan S, Lee HP, Win T, Balakrishnan S. The impact of intrathecal baclofen on the ability to walk: A systematic review. Clin Rehabil. 2023;37(4):462-77.

243. Lee M, Li H, Wasuwanich P, Kim S, Kim J, Jeong G, et al. COVID-19 susceptibility and clinical outcomes in inflammatory bowel disease: An updated systematic review and meta-analysis. Rev Med Virol. 2023;33(2):e2414.

244. Lerman S, Owens M, Liu T, Puthumana J, Hultman C, Caffrey J, et al. Sleep after burn injuries: A systematic review and meta-analysis. Sleep Med Rev. 2022;65.

245. Leutenegger V, Grylka-Baeschlin S, Wieber F, Daly D, Pehlke-Milde J, Leutenegger V, et al. The effectiveness of skilled breathing and relaxation techniques during antenatal education on maternal and neonatal outcomes: a systematic review. BMC Pregnancy Childbirth. 2022;22(1).

246. Li G, Wang R, Zhang C, Li L, Zhang J, Sun G, et al. Consumption of Non-Nutritive Sweetener during Pregnancy and Weight Gain in Offspring: Evidence from Human Studies. Nutrients. 2022;14(23).

247. Li K, Chen S, Wang C, Yang L, Li K-p, Chen S-y, et al. Perioperative and oncologic outcomes of single-port versus conventional robotic-assisted partial nephrectomy: an evidence-based analysis of comparative outcomes. J Robot Surg. 2023;17(3):765-77.

248. Li K, Chen S, Yang L, Li K-p, Chen S-y, Yang L. Laparoscopic simple prostatectomy versus robot-assisted simple prostatectomy for large benign prostatic hyperplasia: a systematic review and meta-analysis of comparative trials. J Robot Surg. 2023;17(2):351-64.

249. Li M, Zhu H, Liu Y, Lu Y, Sun M, Zhang Y, et al. Role of Traditional Chinese Medicine in Treating Severe or Critical COVID-19: A Systematic Review of Randomized Controlled Trials and Observational Studies. Front Pharmacol. 2022;13.

250. Li Y, He X, Li Z, Li D, Yuan X, Yang J, et al. Double sequential external defibrillation versus standard defibrillation in refractory ventricular fibrillation: A systematic review and meta-analysis. Front Cardiovasc Med. 2022;9.

251. Liaghat B, Pedersen JR, Husted RS, Pedersen LL, Thorborg K, Juhl CB. Diagnosis, prevention and treatment of common shoulder injuries in sport: grading the evidence - a statement paper commissioned by the Danish Society of Sports Physical Therapy (DSSF). Br J Sports Med. 2023;57(7):408-16.

252. Liao Z, Zhang Z, Liu Q, Liao Z-M, Zhang Z-M, Liu Q. Hydroxychloroquine/chloroquine and the risk of acute kidney injury in COVID-19 patients: a systematic review and meta-analysis. Ren Fail. 2022;44(1):415-25.

253. Lim Y, Wang Y, Estee M, Abidi J, Kumar M, Hussain S, et al. Metformin as a potential disease-modifying drug in osteoarthritis: a systematic review of pre-clinical and human studies. Osteoarthritis Cartilage. 2022;30(11):1434-42.

254. Lin C, Chiang M, Tam K, Lin C-W, Chiang M-H, Tam K-W. Treatment of Mammary Paget Disease: A systematic review and meta-analysis of real-world data. Int J Surg. 2022;107.

255. Lin H, Salech F, Lim A, Vogrin S, Duque G, Lin H, et al. The effect of rapamycin and its analogues on age-related musculoskeletal diseases: a systematic review. Aging Clin Exp Res. 2022;34(10):2317-33.

256. Lin H, Xu D, Yang M, Ma X, Yan N, Chen H, et al. Behaviour change techniques that constitute effective planning interventions to improve physical activity and diet behaviour for people with chronic conditions: a systematic review. BMJ Open. 2022;12(8).

257. Lin L, Xu Q, Xu F, Zhou C, Huang X, Chen R, et al. Comparison of short-term surgical outcomes and post-operative recovery between single-incision and multi-port laparoscopic distal gastrectomy for gastric cancer. J Minimal Access Surg. 2022;18(4):578-84.

258. Ling S, Ming L, Dhaliwal J, Gupta M, Ardianto C, Goh K, et al. Role of Immunotherapy in the Treatment of Cancer: A Systematic Review. Cancers. 2022;14(21).

259. Lisan Q, Baudouin R, Lechien J, Hans S, Blumen M, Lisan Q, et al. Is drug-induced sleep endoscopy associated with better outcomes after soft tissue surgery for sleep apnea? A systematic review and meta-analysis. Clin Otolaryngol. 2023;48(2):122-9.

260. Liu P, Tan J, Song Y, Huang K, Zhang Q, Xie H, et al. The Application of Magnetic Nanoparticles for Sentinel Lymph Node Detection in Clinically Node-Negative Breast Cancer Patients: A Systemic Review and Meta-Analysis. Cancers. 2022;14(20).

261. Liu S, Silikas N, EI-Angbawi A, Liu S, Silikas N, EI-Angbawi A. Analysis of the effectiveness of the fiber-reinforced composite lingual retainer: A systematic review and meta-analysis. Am J Orthod Dentofac Orthop. 2022;162(5):601-+.

262. Liu X, Yue J, Pervaiz R, Zhang H, Wang L, Liu X, et al. Association between fertility treatments and breast cancer risk in women with a family history or BRCA mutations: a systematic review and meta-analysis. Front Endocrinol. 2022;13.

263. Llewellyn D, Ellis H, Aylwin S, Ostarijas E, Green S, Sheridan W, et al. The efficacy of GLP-1RAs for the management of postprandial hypoglycemia following bariatric surgery: a systematic review. Obesity. 2023;31(1):20-30.

264. Lonati C, Esperto F, Scarpa R, Papalia R, Gomes Rivas J, Alvarez-Maestro M, et al. Bladder perforation during transurethral resection of the bladder: a comprehensive algorithm for diagnosis, management and follow-up. Minerva Urol Nephrol. 2022;74(5):570-80.

265. Lopinto J, Gendreau S, Berti E, Bartolucci P, Habibi A, Dessap A, et al. Effects of corticosteroids in patients with sickle cell disease and acute complications: a systematic review and meta-analysis. Haematologica. 2022;107(8):1914-21.

266. Loyd C, Zhang Y, Weisberg T, Boyett J, Huckaby E, Grundhoefer J, et al. A systematic review and meta-analysis: Assessment of hospital walking programs among older patients. Nurs Open. 2023;10(4):1942-53.

267. Lucena-Anton D, Fernandez-Lopez J, Pacheco-Serrano A, Garcia-Munoz C, Moral-Munoz J, Lucena-Anton D, et al. Virtual and Augmented Reality versus Traditional Methods for Teaching Physiotherapy: A Systematic Review. Eur J Invest Health Psychol Educ. 2022;12(12):1780-92.

268. Lucia S, Alessandro P, Giulia B, Giada F, Massimo D, Daniele B, et al. The bone lid technique in lateral sinus lift: a systematic review and meta-analysis. Int J Implant Dent. 2022;8(1).

269. Luo J, Yu J, Sui Z, Zhong Y, Zheng Q, Li L, et al. Comparison on the effect of seven drugs to prevent relapses of neuromyelitis optica spectrum disorders: A modeling analysis of literature aggregate data. Int Immunopharmacol. 2022;110.

270. Luxton T, King N, Walti C, Jeuken L, Sandoe J, Luxton TN, et al. A Systematic Review of the Effect of Therapeutic Drug Monitoring on Patient Health Outcomes during Treatment with Carbapenems. Antibiotics-Basel. 2022;11(10).

271. Ma N, Gogos S, Moaveni A, Ma N, Gogos S, Moaveni A. Do Intrawound Antibiotics Reduce the Incidence of Surgical Site Infections in Pelvic and Lower-Limb Trauma Surgery? A Systematic Review and Meta-analysis. J Orthop Trauma. 2022;36(11):E418-E24.

272. Majlessi A, Burton J, March D, Majlessi A, Burton JO, March DS. The effect of extended hemodialysis on nutritional parameters: a systematic review. J Nephrol. 2022;35(8):1985-99.

273. Manosroi W, Danpanichkul P, Atthakomol P, Manosroi W, Danpanichkul P, Atthakomol P. Effect of sodium-glucose cotransporter-2 inhibitors on aldosterone and renin levels in diabetes mellitus type 2 patients: a systematic review and meta-analysis. Sci Rep. 2022;12(1).

274. Manouchehri M, Fernandez-Alfonso M, Gil-Ortega M, Manouchehri M, Fernandez-Alfonso MS, Gil-Ortega M. Impact of intervention of community pharmacists on cardiovascular outcomes in Spain: A systematic review. J Pharm Pharmacogn Res. 2022;10(5):952-76.

275. Marcec R, Dodig V, Radanovic I, Likic R, Marcec R, Dodig VM, et al. Intravenous immunoglobulin (IVIg) therapy in hospitalised adult COVID-19 patients: A systematic review and meta-analysis. Rev Med Virol. 2022;32(6).

276. Marini S, Mauro M, Grigoletto A, Toselli S, Latessa P, Marini S, et al. The Effect of Physical Activity Interventions Carried Out in Outdoor Natural Blue and Green Spaces on Health Outcomes: A Systematic Review. Int J Environ Res Public Health. 2022;19(19).

277. Marques D, Neiva H, Marinho D, Marques M, Marques DL, Neiva HP, et al. Manipulating the Resistance Training Volume in Middle-Aged and Older Adults: A Systematic Review with Meta-Analysis of the Effects on Muscle Strength and Size, Muscle Quality, and Functional Capacity. Sports Med. 2023;53(2):503-18.

278. Martimbianco A, Moreira R, Pacheco R, Latorraca C, dos Santos A, Logullo P, et al. Efficacy and safety of hemodialysis strategies for pregnant women with chronic kidney disease: Systematic review. Semin Dial. 2023;36(1):3-11.

279. Marupuru S, Roether A, Guimond A, Stanley C, Pesqueira T, Axon D, et al. A Systematic Review of Clinical Outcomes from Pharmacist Provided Medication Therapy Management (MTM) among Patients with Diabetes, Hypertension, or Dyslipidemia. Healthcare. 2022;10(7).

280. Masson W, Barbagelata L, Lobo M, Nogueira J, Corral P, Lavalle-Cobo A, et al. Effect of Yerba Mate (Ilex paraguariensis) on Lipid Levels: A Systematic Review and Meta-Analysis. Plant Food Hum Nutr. 2022;77(3):353-66.

281. Matsuzaki S, Ueda Y, Matsuzaki S, Nagase Y, Kakuda M, Lee M, et al. Assisted Reproductive Technique and Abnormal Cord Insertion: A Systematic Review and Meta-Analysis. Biomedicines. 2022;10(7).

282. Matthaios S, Tsolakis A, Haidich A, Galanis I, Tsolakis I, Matthaios S, et al. Dental and Skeletal Effects of Herbst Appliance, Forsus Fatigue Resistance Device, and Class II Elastics-A Systematic Review and Meta-Analysis. J Clin Med. 2022;11(23).

283. Mattison G, Canfell O, Forrester D, Dobbins C, Smith D, Toyras J, et al. The Influence of Wearables on Health Care Outcomes in Chronic Disease: Systematic Review. J Med Internet Res. 2022;24(7).

284. Mazurek M, Murray A, Heitman S, Ruan Y, Antoniou S, Boyne D, et al. Association Between Endoscopist Specialty and Colonoscopy Quality: A Systematic Review and Meta-analysis. Clin Gastroenterol Hepatol. 2022;20(9):1931-46.

285. Mazzetti T, Santos P, Antunes H, Montagner A, van de Sande F, Maske T, et al. Required time for pre-oncological dental management - A rapid review of the literature. Oral Oncol. 2022;134.

286. McClelland G, Hepburn S, Finch T, Price C, McClelland G, Hepburn S, et al. How do interventions to improve the efficiency of acute stroke care affect prehospital times? A systematic review and narrative synthesis. BMC Emerg Med. 2022;22(1).

287. McNair C, Chirinian N, Uleryk E, Stevens B, McAllister M, Franck L, et al. Effectiveness of parental education about pain in the neonatal period on knowledge, attitudes, and practices: A systematic review and meta-analysis. Paediatr Child Health. 2022;27(8):454-63.

288. Media A, Rajendran R, Kimose H, El-Akkawi A, Media AS, Rajendran R, et al. Effect of preoperative ultrasound mapping of the saphenous vein on leg wound complications after coronary artery bypass surgery: a systematic review. Cardiothorac Surgeon. 2022;30(1).

289. Meggiolaro A, Schepisi M, Farina S, Castagna C, Mammone A, Siddu A, et al. Effectiveness of vaccination against SARS-CoV-2 Omicron variant infection, symptomatic disease, and hospitalization: a systematic review and meta-analysis. Expert Rev Vaccines. 2022;21(12):1831-41.

290. Meng C, Gan L, Li K, Peng L, Li J, Yang J, et al. Comparison of external stents and DJ stents techniques for pediatric pyeloplasty: A systematic review and meta-analysis. Front Pediatr. 2022;10.

291. Michelland L, Murad M, Bougouin W, Van der Broek M, Prokop L, Anys S, et al. Association between basic life support and survival in sports-related sudden cardiac arrest: a meta-analysis( ). Eur Heart J. 2023;44(3):180-92.

292. Mifsud T, Modestini C, Mizzi A, Falzon O, Cassar K, Mizzi S, et al. The Effects of Skin Temperature Changes on the Integrity of Skin Tissue: A Systematic Review. Adv Skin Wound Care. 2022;35(10):555-65.

293. Milioglou I, Farmakis I, Wazirali M, Ajluni S, Khawaja T, Chatuverdi A, et al. Percutaneous thrombectomy in patients with intermediate- and high-risk pulmonary embolism and contraindications to thrombolytics: a systematic review and meta-analysis. J Thromb Thrombolysis. 2023;55(2):228-42.

294. Milone M, Gallo G, Grossi U, Pelizzo P, D'Amore A, Manigrasso M, et al. Endoscopic sinusectomy: ‘a rose by any other name’. A systematic review of different endoscopic procedures to treat pilonidal disease. Colorectal Dis. 2023;25(2):177-90.

295. Mina D, Tandon P, Kow A, Chan A, Edbrooke L, Raptis D, et al. The role of acute in-patient rehabilitation on short-term outcomes after liver transplantation: A systematic review of the literature and expert panel recommendations. Clin Transplant. 2022;36(9).

296. Miranda V, Dal Pizzol T, de Jesus P, Silveira M, Bertoldi A, Avena Miranda VI, et al. Iron Salts, High Levels of Hemoglobin and Ferritin in Pregnancy, and Development of Gestational Diabetes: A Systematic Review. Rev Bras Ginecol Obstet. 2022;44(11):1059-69.

297. Mirshahi A, Khalilipour E, Faghihi H, Riazi-Esfahani H, Mirshahi R, Mehrjardi H, et al. Pars plana vitrectomy combined with phacoemulsification versus pars plana vitrectomy only for treatment of phakic rhegmatogenous retinal detachment: a systematic review and meta-analysis. Int Ophthalmol. 2023;43(2):697-706.

298. Moffatt S, Venturini S, Vulliamy P, Moffatt S, Venturini S, Vulliamy P. Does pre-injury clopidogrel use increase the risk of intracranial haemorrhage post head injury in adult patients? A systematic review and meta-analysis. Emerg Med J. 2023;40(3):175-81.

299. Mohan K, Sivarajan S, Lau M, Othman S, Fayed M, Mohan K, et al. Soft tissue changes with skeletal anchorage in comparison to conventional anchorage protocols in the treatment of bimaxillary proclination patients treated with premolar extraction A systematic review. J Orofac Orthop. 2024;85(2):146-62.

300. Moldovan R, Mester A, Piciu A, Bran S, Onisor F, Moldovan R, et al. Clinical Outcomes of Enamel Matrix Derivate Used in Surgical and Non-Surgical Treatment of Peri-Implantitis: A Systematic Review of Clinical Studies. Med Lith. 2022;58(12).

301. Montrucchio G, Balzani E, Lombardo D, Giaccone A, Vaninetti A, D'Antonio G, et al. Proadrenomedullin in the Management of COVID-19 Critically Ill Patients in Intensive Care Unit: A Systematic Review and Meta-Analysis of Evidence and Uncertainties in Existing Literature. J Clin Med. 2022;11(15).

302. Moreira H, Sousa J, Mansilha A, Moreira H, Sousa J, Mansilha A. Chemothromboprophylaxis in varicose vein surgery: a systematic review. Int Angiol. 2022;41(4):346-55.

303. Morris T, Aspinal F, Ledger J, Li K, Gomes M, Morris T, et al. The Impact of Digital Health Interventions for the Management of Type 2 Diabetes on Health and Social Care Utilisation and Costs: A Systematic Review. Pharmacoecon-Open. 2023;7(2):163-73.

304. Muna Al Mushaikhi JTBD, Amelia S. Health Education, its Methods and Effects on Parents’ Knowledge, Attitudes, and Behaviours to Prevent Unintentional Child Injuries at Home: A Systematic Review. Child Care Pract. 2022;0(0):1--26.

305. Murtha-Lemekhova A, Fuchs J, Hoffmann K, Murtha-Lemekhova A, Fuchs J, Hoffmann K. Innovation for the Sake of Innovation? How Does Robotic Hepatectomy Compare to Laparoscopic or Open Resection for HCC-A Systematic Review and Meta-Analysis. Cancers. 2022;14(14).

306. Mutabazi J, Werfalli M, Rawat A, Musa E, Chivese T, Norris S, et al. Integrated Management of Type 2 Diabetes and Gestational Diabetes in the Context of Multi-Morbidity in Africa: A Systematic Review. Int J Integr Care. 2022;22(3).

307. Nag A, Saltagi A, Saltagi M, Wu A, Higgins T, Knisely A, et al. Management of Post-Infectious Anosmia and Hyposmia: A Systematic Review. Ann Otol Rhinol Laryngol. 2023;132(7):806-17.

308. Neris Almeida Viana S, do Reis Santos Pereira T, de Carvalho Alves J, Tianeze de Castro C, Santana CdSL, Henrique Sousa Pinheiro L, et al. Benefits of probiotic use on COVID-19: A systematic review and meta-analysis. Crit Rev Food Sci Nutr. 2024;64(10):2986-98.

309. Ng W, But B, Wong C, Choi C, Chua M, Blanchard P, et al. Particle beam therapy for nasopharyngeal cancer: A systematic review and meta-analysis. Clin Transl Radiat Oncol. 2022;37:41-56.

310. Nichani P, Dhoot A, Popovic M, Eshtiaghi A, Mihalache A, Sayal A, et al. Scleral Buckling Alone or in Combination with Pars Plana Vitrectomy for Rhegmatogenous Retinal Detachment Repair: A Meta-Analysis of 7,212 Eyes. Ophthalmologica. 2022;245(4):296-314.

311. Nie J, Karras C, Texakalidis P, Trybula S, Dahdaleh N, Nie JZ, et al. A Systematic Review of Outpatient Telemedicine Use in Neurosurgery Since the Start of Coronavirus Disease 2019. World Neurosurg. 2022;167:E1090-E102.

312. Niforatos J, Ehmann M, Balhara K, Hinson J, Ramcharran L, Lobner K, et al. Management of atrial flutter and atrial fibrillation with rapid ventricular response in patients with acute decompensated heart failure: A systematic review. Acad Emerg Med. 2023;30(2):124-32.

313. Niu H, Atallah E, Alvarez-Alvarez I, Medina-Caliz I, Aithal G, Arikan C, et al. Therapeutic Management of Idiosyncratic Drug-Induced Liver Injury and Acetaminophen Hepatotoxicity in the Paediatric Population: A Systematic Review. Drug Saf. 2022;45(11):1329-48.

314. Nochaiwong S, Ruengorn C, Awiphan R, Chai-Adisaksopha C, Tantraworasin A, Phosuya C, et al. Use of serotonin reuptake inhibitor antidepressants and the risk of bleeding complications in patients on anticoagulant or antiplatelet agents: a systematic review and meta-analysis. Ann Med. 2022;54(1):80-97.

315. Nolde M, Ahn N, Dreischulte T, Krause E, Guntner F, Gunter A, et al. Proton pump inhibitors and the risk of cardiovascular events and cardiovascular mortality: A systematic review and meta-analysis of observational studies. Eur J Intern Med. 2022;106:80-9.

316. Obaid S, Chen J, Ibrahim G, Bouthillier A, Dimentberg E, Surbeck W, et al. Predictors of outcomes after surgery for medically intractable insular epilepsy: A systematic review and individual participant data meta-analysis. Epilepsia Open. 2023;8(1):12-31.

317. Oderkerk T, van de Kar M, Cornel K, Bongers M, Geomini P, Oderkerk TJ, et al. Endometrial cancer after endometrial ablation: a systematic review. Int J Gynecol Cancer. 2022;32(12):1555-60.

318. Oikonomidi T, Norman G, McGarrigle L, Stokes J, van der Veer S, Dowding D, et al. Predictive model-based interventions to reduce outpatient no-shows: a rapid systematic review. J Am Med Inf Assoc. 2023;30(3):559-69.

319. Olczak-Pruc M, Swieczkowski D, Ladny J, Pruc M, Juarez-Vela R, Rafique Z, et al. Vitamin C Supplementation for the Treatment of COVID-19: A Systematic Review and Meta-Analysis. Nutrients. 2022;14(19).

320. Oppici L, Stell F, Utesch T, Woods C, Foweather L, Rudd J, et al. A Skill Acquisition Perspective on the Impact of Exergaming Technology on Foundational Movement Skill Development in Children 3-12 Years: A Systematic Review and Meta-analysis. Sports Med-Open. 2022;8(1).

321. Opris H, Baciut M, Bran S, Onisor F, Almasan O, Manea A, et al. Lateral Cephalometric Analytical Uses for Temporomandibular Joint Disorders: The Importance of Cervical Posture and Hyoid Position. Int J Environ Res Public Health. 2022;19(17).

322. Ost L, Enebrink P, Finnes A, Ghaderi A, Havnen A, Kvale G, et al. Cognitive behavior therapy for obsessive-compulsive disorder in routine clinical care: A systematic review and meta-analysis. Behav Res Ther. 2022;159.

323. Ostapenko E, Nixdorf L, Devyatko Y, Exner R, Wimmer K, Fitzal F, et al. Prepectoral Versus Subpectoral Implant-Based Breast Reconstruction: A Systemic Review and Meta-analysis. Ann Surg Oncol. 2023;30(1):126-36.

324. Oswald D, Pallauf M, Deininger S, Torzsok P, Sieberer M, Eiben C, et al. Neoadjuvant Chemotherapy before Nephroureterectomy in High-Risk Upper Tract Urothelial Cancer: A Systematic Review and Meta-Analysis. Cancers. 2022;14(19).

325. Ozawa Y, Miyake F, Isayama T, Ozawa Y, Miyake F, Isayama T. Efficacy and safety of permissive hypercapnia in preterm infants: A systematic review. Pediatr Pulmonol. 2022;57(11):2603-13.

326. Pan Y, Xu M, Kang J, Wang S, Liu X, Pan Y, et al. The Safety of Continuing Low-Dose Aspirin Therapy Perioperatively in Percutaneous Nephrolithotomy: A Systematic Review and Meta-analysis. Urol J. 2022;19(4):253-61.

327. Pang N, Ng C, Wong C, Pang NQ, Ng CSY, Wong CJH. Laparoscopic versus open groin hernia repair in older adults: a systematic review and meta-analysis. ANZ J Surg. 2022;92(10):2457-63.

328. Park S, Kim S-H. A systematic review and meta-analysis of bystander intervention programs for intimate partner violence and sexual assault. Psychol Violence. 2023;13(2):93-106.

329. Parker K, Hartemink J, Saha A, Mitra R, Lewis P, Power A, et al. A systematic review of the efficacy and safety of anticoagulants in advanced chronic kidney disease. J Nephrol. 2022;35(8):2015-33.

330. Partap U, Chowdhury R, Taneja S, Bhandari N, De Costa A, Bahl R, et al. Preconception and periconception interventions to prevent low birth weight, small for gestational age and preterm birth: a systematic review and meta-analysis. BMJ Glob Health. 2022;7(8).

331. Pathak R, Kang D, Lu Y, Mansuri F, Kasen S, Deng Y, et al. Should we abandon annual physical examination?-A meta-analysis of annual physical examination and all-cause mortality in adults based on observational studies. Prev Med. 2022;161.

332. Patil A, Shaikh S, Karajgi A, Ali M, Patil A, Shaikh SZ, et al. Efficacy and safety of botulinum toxin-A in writer's cramp: a systematic review, meta-analysis, and meta-regression. Egypt J Neurol Psychiatr Neurosurg. 2022;58(1).

333. Patterson K, Davey R, Keegan R, Kunstler B, Woodward A, Freene N, et al. Behaviour change techniques in cardiovascular disease smartphone apps to improve physical activity and sedentary behaviour: Systematic review and meta-regression. Int J Behav Nutr Phys Act. 2022;19(1).

334. Paulussen E, Mulder F, Mathijssen N, Witlox M, Paulussen EMB, Mulder FECM, et al. Active monitoring versus immediate abduction as treatment of stable developmental dysplasia of the hip: a systematic review of the literature. BMJ Open. 2022;12(9).

335. Peers E, Boocock R, Burn N, Peers E, Boocock RC, Burn N. A systematic review examining the impact of blended diets on the gastrointestinal symptoms of people who are enterally fed. J Hum Nutr Diet. 2023;36(3):673-86.

336. Peiyao R, Mengjie Y, Xiaogang S, Wenfang H, Danna Z, Yuqun Z, et al. Immunogenicity and safety of SARS-CoV-2 vaccine in hemodialysis patients: A systematic review and meta-analysis. Front Public Health. 2022;10.

337. Peng J, Li S, Lin X, Zhong D, Zheng R, Huang M, et al. Anterior cervical discectomy and fusion without plate (ACDFWP) versus anterior cervical disc arthroplasty (ACDA) for cervical spondylosis: A meta-analysis and literature review. Intractable Rare Dis Res. 2022;11(3):105-12.

338. Peng M, Xiao T, Carter B, Chen P, Shearer J, Peng M, et al. Effectiveness and Cost-Effectiveness of Mental Health Interventions Delivered by Frontline Health Care Workers in Emergency Health Services: A Systematic Review and Meta-Analysis. Int J Environ Res Public Health. 2022;19(23).

339. Perez-Camargo D, Travieso A, Carnero-Alcazar M, Taramasso M, Cobiella-Carnicer J, Maroto-Castellanos L, et al. Neurological outcomes of transcatheter aortic valve implantation with or without cerebral embolic protection devices: A meta-analysis. J Stroke Cerebrovasc Dis. 2022;31(9).

340. Pham X, Ray J, Neto A, Laing O, Perucca P, Kwan P, et al. Association of Neurocritical Care Services With Mortality and Functional Outcomes for Adults With Brain Injury A Systematic Review and Meta-analysis. JAMA Neurol. 2022;79(10):1049-58.

341. Piragine E, Petri D, Giometto S, Martelli A, Lucenteforte E, Calderone V, et al. Potential effects of Alliaceae and Brassicaceae edible plants on blood glucose levels in patients with type 2 diabetes: A systematic review and meta-analysis of clinical trials. Pharmacol Res. 2022;185.

342. Pitre T, Kiflen M, Ho T, Seijo L, Zeraatkar D, de Torres J, et al. Inhaled corticosteroids, COPD, and the incidence of lung cancer: a systematic review and dose response meta-analysis. BMC Pulm Med. 2022;22(1).

343. Pitsios C, Vassilopoulou E, Pantavou K, Terreehorst I, Nowak-Wegzryn A, Cianferoni A, et al. Allergy-Test-Based Elimination Diets for the Treatment of Eosinophilic Esophagitis: A Systematic Review of Their Efficacy. J Clin Med. 2022;11(19).

344. Ponugoti N, Raghu A, Kosy J, Magill H, Ponugoti N, Raghu A, et al. A comparison of distal femoral replacement versus fixation in treating periprosthetic supracondylar femur fractures: a systematic review and meta-analysis. Arch Orthop Trauma Surg. 2023;143(6):3335-45.

345. Pouliezou I, Xenou A, Vavetsi K, Mitsea A, Sifakakis I, Pouliezou I, et al. Adverse Effects of Surgically Accelerated Orthodontic Techniques: A Systematic Review. Children-Basel. 2022;9(12).

346. Poupore N, Chen T, Nguyen S, Nathan C, Newman J, Poupore NS, et al. Transoral Robotic Surgery for Oropharyngeal Squamous Cell Carcinoma of the Tonsil versus Base of Tongue: A Systematic Review and Meta-Analysis. Cancers. 2022;14(15).

347. Poupore N, Gordis T, Nguyen S, Meyer T, Carroll W, Lambert P, et al. Tympanoplasty With and Without Mastoidectomy for Chronic Otitis Media Without Cholesteatoma: A Systematic Review and Meta-analysis. Otol Neurotol. 2022;43(8):864-73.

348. Poupore N, Smaily H, Carroll W, Pecha P, Poupore NS, Smaily H, et al. Outcomes of Tympanoplasty After Cleft Palate Repair: A Systematic Review and Meta-analysis. Otolaryngol Head Neck Surg. 2023;169(1):1-11.

349. Prayogo S, Andrew H, Cong S, Intaran K, Prayogo SA, Andrew H, et al. Photodynamic therapy in the treatment of condyloma acuminata: A systematic review of clinical trials. Int J STD AIDS. 2023;34(2):76-86.

350. Prokopidis K, Kirwan R, Giannos P, Triantafyllidis K, Kechagias K, Forbes S, et al. The impact of branched-chain amino acid supplementation on measures of glucose homeostasis in individuals with hepatic disorders: A systematic review of clinical studies. J Hum Nutr Diet. 2023;36(3):603-11.

351. Psaltis E, Varghese C, Pandanaboyana S, Nayar M, Psaltis E, Varghese C, et al. Quality of life after surgical and endoscopic management of severe acute pancreatitis: A systematic review. World J Gastrointest Endosc. 2022;14(7):443-54.

352. Pugalendhi R, Jayaprakasan N, Karuveettil V, Varma N, Ajith V, Prabha R, et al. Effectiveness of intraoral scanners in full arch digital impression - a systematic review. Clin Invest Orthodontics. 2022;81(3):127-36.

353. Punj E, Collins A, Agravedi N, Marriott J, Sapey E, Punj E, et al. What is the evidence that a pharmacy team working in an acute or emergency medicine department improves outcomes for patients: A systematic review. Pharmacol Res Perspect. 2022;10(5).

354. Qian N, Xu J, Wang Y, Qian N, Xu J, Wang Y. Stroke Risks in Primary Aldosteronism with Different Treatments: A Systematic Review and Meta-Analysis. J Cardiovasc Dev Dis. 2022;9(9).

355. Qiu Q, Chen S, Qiu Y, Mao W, Qiu Q, Chen S, et al. Cardiac Shock Wave Therapy in Coronary Artery Disease: A Systematic Review and Meta-Analysis. Front Cardiovasc Med. 2022;9.

356. Raghis T, Alsulaiman T, Mahmoud G, Youssef M, Raghis TR, Alsulaiman TMA, et al. Efficiency of maxillary total arch distalization using temporary anchorage devices (TADs) for treatment of Class II-malocclusions: A systematic review and meta-analysis. Int Orthod. 2022;20(3).

357. Ramaswamy V, de Almeida M, Dawson J, Trevisanuto D, Nakwa F, Kamlin C, et al. Review Maintaining normal temperature immediately after birth in late preterm and term infants: A systematic review and meta-analysis. Resuscitation. 2022;180:81-98.

358. Ranganath K, Jalisi S, Naples J, Gomez E, Ranganath K, Jalisi SM, et al. Comparing outcomes of radial forearm free flaps and anterolateral thigh free flaps in oral cavity reconstruction: A systematic review and meta-analysis. Oral Oncol. 2022;135.

359. Rehman Y, Kirsch J, Wang M, Ferguson H, Bingham J, Senger B, et al. Impact of osteopathic manipulative techniques on the management of dizziness caused by neuro-otologic disorders: systematic review and meta-analysis. J Osteopath Med. 2023;123(2):91-101.

360. Reilly EB, Stuyvenberg CL. A Meta-analysis of Loving-Kindness Meditations on Self-Compassion. Mindfulness. 2023;14(10):2299-310.

361. Reiter A, De Meulemeester J, Kenya-Mugisha N, Tagoola A, Kabajaasi O, Wiens M, et al. Parental participation in the care of hospitalized neonates in low- and middle-income countries: A systematic review and meta-analysis. Front Pediatr. 2022;10.

362. Rinaldi M, Bonazzetti C, Gatti M, Caroccia N, Comai G, Ravaioli M, et al. The impact of preservation fluid culture on graft site arteritis: A systematic review and meta-analysis. Transpl Infect Dis. 2022;24(6):e13979.

363. Robinson A, Kennedy L, Roper T, Khan M, Jaunoo S, Robinson A, V, et al. The management of chyle leak post-oesophagectomy for oesophageal carcinoma: a systematic review. Ann R Coll Surg Engl. 2022;104(7):480-9.

364. Romer C, Czupajllo J, Wolfarth B, Lerchbaumer M, Legerlotz K, Roemer C, et al. Effects of orally administered hormonal contraceptives on the musculoskeletal system of healthy premenopausal women-A systematic review. Health Sci Rep. 2022;5(5).

365. Romero D, Escolano J, Fernandez C, Martinez-Toldos J, Monera C, Castilla G, et al. The influence of the Artisan-Verisyse position on the postoperative outcomes: A systematic review and meta-analysis. Indian J Ophthalmol. 2022;70(9):3213-21.

366. Roohani S, Ehret F, Kobus M, Florcken A, Mardian S, Striefler J, et al. Preoperative hypofractionated radiotherapy for soft tissue sarcomas: a systematic review. Radiat Oncol. 2022;17(1).

367. Rungjirajittranon T, Owattanapanich W, Chinthammitr Y, Ruchutrakool T, Suwanawiboon B, Rungjirajittranon T, et al. Direct oral anticoagulants versus low-molecular-weight heparins for the treatment of acute venous thromboembolism in patients with gastrointestinal cancer: a systematic review and meta-analysis. Thromb J. 2022;20(1).

368. Rustia S, Lam J, Tahir P, Al Kharafi L, Oberoi S, Ganguly R, et al. Three-dimensional morphologic changes in the temporomandibular joint in asymptomatic patients who undergo orthodontic treatment: A systematic review. Oral Surg Oral Med Oral Pathol Oral Radiol. 2022;134(3):397-406.

369. Ryan E, Creaven A, Neill E, O'Suilleabhain P, Ryan EM, Creaven A-M, et al. Anxiety Following Myocardial Infarction: A Systematic Review of Psychological Interventions. Health Psychol. 2022;41(9):599-610.

370. Sa M, Jacquemyn X, Tasoudis P, Van den Eynde J, Erten O, Dokollari A, et al. Immediate and late outcomes of transcatheter aortic valve implantation versus surgical aortic valve replacement in bicuspid valves: Meta-analysis of reconstructed time-to-event data. J Card Surg. 2022;37(10):3300-10.

371. Sa M, Jacquemyn X, Tasoudis P, Van den Eynde J, Erten O, Sicouri S, et al. Long-term outcomes of total arch replacement versus proximal aortic replacement in acute type A aortic dissection: Meta-analysis of Kaplan-Meier-derived individual patient data. J Card Surg. 2022;37(12):4256-66.

372. Sack D, Peetluk L, Audet C, Sack DE, Peetluk LS, Audet CM. Couples-based interventions and postpartum contraceptive uptake: A systematic review. Contraception. 2022;112:23-36.

373. Safavi D, Creavin B, Gallagher T, Kelly M, Safavi D, Creavin B, et al. The role of bariatric surgery in liver transplantation: timing and type. Langenbecks Arch Surg. 2022;407(8):3249-58.

374. Sarwar M, McDonald V, Abramson M, McLoughlin R, Geethadevi G, George J, et al. Effectiveness of Interventions Targeting Treatable Traits for the Management of Obstructive Airway Diseases: A Systematic Review and Meta-Analysis. J Allergy Clin Immunol-Pract. 2022;10(9):2333-+.

375. Saunders S, Sutcliffe K, McOrist N, Levett K, Saunders SL, Sutcliffe KL, et al. The associations between women who are immigrants, refugees, or asylum seekers, access to universal healthcare, and the timely uptake of antenatal care: A systematic review. Aust N Z J Obstet Gynaecol. 2023;63(2):134-45.

376. Scarcella S, Law Y, Bravi C, Piazza P, Heldwein F, Ronchi P, et al. Does using a laser improve outcomes of conventional circumcision in adult and children populations? Results from a systematic review and meta-analysis. Andrology. 2023;11(1):54-64.

377. Schlacter J, Kay-Rivest E, Nicholson J, Santacatterina M, Zhang Y, Jethanamest D, et al. Cochlear Implantation Outcomes in Patients With Retrocochlear Pathology: A Systematic Review and Pooled Analysis. Otol Neurotol. 2022;43(9):980-6.

378. Scognamiglio P, Reeh M, Melling N, Kantowski M, Eichelmann A, Chon S, et al. Management of intra-thoracic anastomotic leakages after esophagectomy: updated systematic review and meta-analysis of endoscopic vacuum therapy versus stenting. BMC Surg. 2022;22(1).

379. Scott L, Wilson R, Davies P, Lyttle M, Mytton J, Dawson S, et al. Educational interventions to prevent paediatric abusive head trauma in babies younger than one year old: A systematic review and meta-analyses. Child Abuse Negl. 2022;134.

380. Scuteri D, Guida F, Boccella S, Palazzo E, Maione S, Rodriguez-Landa J, et al. Effects of Palmitoylethanolamide (PEA) on Nociceptive, Musculoskeletal and Neuropathic Pain: Systematic Review and Meta-Analysis of Clinical Evidence. Pharmaceutics. 2022;14(8).

381. Seda V, Moles R, Carter S, Schneider C, Seda V, Moles RJ, et al. Assessing the comparative effectiveness of implementation strategies for professional services to community pharmacy: A systematic review. Res Soc Adm Pharm. 2022;18(9):3469-83.

382. Sef D, Trkulja V, Raja S, Hooper J, Turina M, Sef D, et al. Comparing mid-term outcomes of Cox-Maze procedure and pulmonary vein isolation for atrial fibrillation after concomitant mitral valve surgery: A systematic review. J Card Surg. 2022;37(11):3801-10.

383. Seidu S, Cebrian A, Kunutsor S, Khunti K, Seidu S, Cebrian A, et al. Erectile dysfunction, phosphodiesterase-5 inhibitor use and risk of cardiovascular disease and mortality in people with diabetes: A systematic review and meta-analysis. Prim Care Diabetes. 2022;16(5):601-13.

384. Sena G, Paglione D, Gallo G, Goglia M, Osso M, Nardo B, et al. Surgical Resection of a Recurrent Hepatocellular Carcinoma with Portal Vein Thrombosis: Is It a Good Treatment Option? A Case Report and Systematic Review of the Literature. J Clin Med. 2022;11(18).

385. Shabanzadeh D, Christensen D, Ewertsen C, Friis-Andersen H, Helgstrand F, Jorgensen L, et al. National clinical practice guidelines for the treatment of symptomatic gallstone disease: 2021 recommendations from the Danish Surgical Society. Scand J Surg. 2022;111(3):11-30.

386. Shah S, Makker K, Zhang M, Harnett S, Aziz K, Hudak M, et al. Dual medication therapy (acetaminophen and ibuprofen) for the management of patent ductus arteriosus in preterm infants: a systematic review and meta-analysis. J Perinatol. 2022;42(12):1654-61.

387. Sharew N, Sharew NT. The Effect of Multimodal Non-pharmacological Interventions on Cognitive Function Improvement for People With Dementia: A Systematic Review. Front Public Health. 2022;10.

388. Shehata M, Ibrahim M, Ghozy S, Bilgin C, Jabal M, Kadirvel R, et al. Long-term outcomes of flow diversion for unruptured intracranial aneurysms: a systematic review and meta-analysis. J NeuroInterventional Surg. 2023;15(9):898-902.

389. Shenoy R, Kirkland P, Hadaya J, Tranfield M, DeVirgilio M, Russell M, et al. Management of symptomatic cholelithiasis: a systematic review. Syst Rev. 2022;11(1).

390. Shieu M, Zaheed A, Shannon C, Chervin R, Conceicao A, Paulson H, et al. Positive Airway Pressure and Cognitive Disorders in Adults With Obstructive Sleep Apnea A Systematic Review of the Literature. Neurology. 2022;99(4):E334-E46.

391. Shin H, Park J, Roh S, Jeon S, Shin HK, Park JH, et al. Meta-Analysis on the Effect of Hypothermia in Acute Spinal Cord Injury. Neurospine. 2022;19(3):748-+.

392. Shipton E, Callaway L, Foxcroft K, Lee N, de Jersey S, Shipton EV, et al. Midwife-Led Continuity of Antenatal Care and Breastfeeding Duration Beyond Postpartum Hospital Discharge: A Systematic Review. J Hum Lact. 2023;39(3):427-40.

393. Shirvani M, Soufi F, Nouralishahi A, Vakili K, Salimi A, Lucke-Wold B, et al. The Diagnostic Value of Neutrophil to Lymphocyte Ratio as an Effective Biomarker for Eye Disorders: A Meta-Analysis. Biomed Res Int. 2022;2022.

394. Shlobin N, Montgomery E, Mohammad L, Kandula V, Beestrum M, DeCuypere M, et al. Visual Outcomes After Treatment for Sporadic Optic Pathway Gliomas in Pediatric Patients: A Systematic Review. World Neurosurg. 2022;164:436-+.

395. Shrestha S, Bhuvan K, Blebil A, Li Teoh S, Shrestha S, Bhuvan KC, et al. Pharmacist Involvement in Cancer Pain Management: A Systematic Review and Meta-Analysis. J Pain. 2022;23(7):1123-42.

396. Simard C, Gerstein L, Cafaro T, Filion K, Douros A, Malhame I, et al. Bleeding in women with venous thromboembolism during pregnancy: A systematic review of the literature. Res Pract Thromb Haemost. 2022;6(6).

397. So B, Jardim L, Schuch L, Kovalski L, Zan R, Calcagnotto T, et al. Analysis of factors that influence quality of life of individuals undergoing treatment for mandibular fractures: A systematic review and meta-analysis. Oral Surg Oral Med Oral Pathol Oral Radiol. 2022;134(3):289-301.

398. Socha P, McGee A, Bhattacharya S, Young C, Wang R, Socha P, et al. Antenatal Corticosteroids and Neonatal Outcomes in Twins A Systematic Review and Meta-analysis. Obstet Gynecol. 2022;140(1):20-30.

399. Sohal S, Mathai S, Nagraj S, Kurpad K, Suthar K, Mehta H, et al. Comparison of Suture-Based and Collagen-Based Vascular Closure Devices for Large Bore Arteriotomies-A Meta-Analysis of Bleeding and Vascular Outcomes. J Cardiovasc Dev Dis. 2022;9(10).

400. Soliman M, Khan A, Azmy S, Gilbert O, Khan S, Goliber R, et al. Meta-analysis of overall survival and postoperative neurologic deficits after resection or biopsy of butterfly glioblastoma. Neurosurg Rev. 2022;45(6):3511-21.

401. Sothivannan A, Eshtiaghi A, Dhoot A, Popovic M, Garg S, Kertes P, et al. Impact of the Time to Surgery on Visual Outcomes for Rhegmatogenous Retinal Detachment Repair: A Meta-Analysis. Am J Ophthalmol. 2022;244:19-29.

402. Soto-Chavez M, Munoz-Velandia O, Alzate-Granados J, Lombo C, Henao-Carrillo D, Gomez-Medina A, et al. Effectiveness and safety of new oral and injectable agents for in-hospital management of type 2 diabetes in general wards: Systematic review and meta-analysis. Diabetes Res Clin Pract. 2022;191.

403. Sousa J, Mendonca D, Teixeira R, Goncalves L, Sousa JP, Mendonca D, et al. Do adrenergic alpha-antagonists increase the risk of poor cardiovascular outcomes? A systematic review and meta-analysis. ESC Heart Fail. 2022;9(5):2823-39.

404. Sprouse L, Liles A, Cronk R, Bauza V, Tidwell JB, Manga M. Interventions to address unsafe child feces disposal practices in the Asia-Pacific region: a systematic review. H2Open J. 2022;5(4):583-602.

405. Starzec-Proserpio M, Bardin M, Fradette J, Tu L, Berube-Lauziere Y, Pare J, et al. High-Intensity Laser Therapy (HILT) as an Emerging Treatment for Vulvodynia and Chronic Musculoskeletal Pain Disorders: A Systematic Review of Treatment Efficacy. J Clin Med. 2022;11(13).

406. Storey B, Verkerk M, Hashtroudi A, Golding-Wood D, Storey B, Verkerk M, et al. A systematic review of interventions to prevent work-related musculoskeletal disorders in ENT surgeons. J Laryngol Otol. 2022;136(7):622-7.

407. Strawbridge R, Javed R, Cave J, Jauhar S, Young A, Strawbridge R, et al. The effects of reserpine on depression: A systematic review. J Psychopharmacol. 2023;37(3):248-60.

408. Strzebonska K, Blukacz M, Wasylewski M, Polak M, Gyawali B, Waligora M, et al. Risk and benefit for umbrella trials in oncology: a systematic review and meta-analysis. BMC Med. 2022;20(1).

409. Szabo G, Szigetvary C, Szabo L, Dembrovszky F, Rottler M, Ocskay K, et al. Point-of-care ultrasound improves clinical outcomes in patients with acute onset dyspnea: a systematic review and meta-analysis. Intern Emerg Med. 2023;18(2):639-53.

410. Taburee W, Dhippayom T, Nagaviroj K, Dilokthornsakul P, Taburee W, Dhippayom T, et al. Effects of Anticholinergics on Death Rattle: A Systematic Review and Network Meta-Analysis. J Palliat Med. 2023;26(3):431-40.

411. Tan T, Luo Y, Hu J, Li F, Fu Y, Tan T, et al. Nonoperative management with angioembolization for blunt abdominal solid organ trauma in hemodynamically unstable patients: a systematic review and meta-analysis. Eur J Trauma Emerg Surg. 2023;49(4):1751-61.

412. Tan V, Peck E, Sivarajah S, Tan W, Ho L, Ng J, et al. Systematic review and meta-analysis of postoperative pain and symptoms control following laser haemorrhoidoplasty versus Milligan-Morgan haemorrhoidectomy for symptomatic haemorrhoids: a new standard. Int J Colorectal Dis. 2022;37(8):1759-71.

413. Tao B, Yang L, Huang Q, Wu J, Liu J, Bei T, et al. Effectiveness of bone substitute materials in opening wedge high tibial osteotomy: a systematic review and meta-analysis. Ann Med. 2022;54(1):565-77.

414. Tasoudis P, Varvoglis D, Tzoumas A, Doulamis I, Tzani A, Sa M, et al. Percutaneous coronary intervention versus coronary artery bypass graft surgery in dialysis-dependent patients: A pooled meta-analysis of reconstructed time-to-event data. J Card Surg. 2022;37(10):3365-73.

415. Taubin D, Berger A, Greenwald D, Greenwald H, Burke C, Gongora D, et al. A systematic review of virtual reality therapies for substance use disorders: Impact on secondary treatment outcomes. Am J Addict. 2023;32(1):13-23.

416. Tejapira K, Yongpisarn T, Sakpuwadol N, Suchonwanit P, Tejapira K, Yongpisarn T, et al. Platelet-rich plasma in alopecia areata and primary cicatricial alopecias: A systematic review. Front Med. 2022;9.

417. Temperley H, O'Sullivan N, Keyes A, Kavanagh D, Larkin J, Mehigan B, et al. Optimal surgical management strategy for treatment of primary anorectal malignant melanoma-a systematic review and meta-analysis. Langenbecks Arch Surg. 2022;407(8):3193-200.

418. Thakar A, Panara K, Goyal M, Kumari R, Sungchol K, Thakar A, et al. AYUSH (Indian System of Medicines) Therapeutics for COVID-19: A Living Systematic Review and Meta-Analysis (First Update). J Integr Complement Med. 2023;29(3):139-55.

419. Thavarajasingam S, El-Khatib M, Vemulapalli K, Iradukunda H, Vishnu K, Borchert R, et al. Radiological predictors of shunt response in the diagnosis and treatment of idiopathic normal pressure hydrocephalus: a systematic review and meta-analysis. Acta Neurochir. 2023;165(2):369-419.

420. Thevarajah A, Wallen M, Imms C, Lonsdale C, Carey J, Froude E, et al. Impact of adapted bicycle riding on outcomes for children and adolescents with disabilities: A systematic review. Dev Med Child Neurol. 2023;65(4):456-68.

421. Thielemann J, Kasparik B, Konig J, Unterhitzenberger J, Rosner R, Thielemann JFB, et al. A systematic review and meta-analysis of trauma-focused cognitive behavioral therapy for children and adolescents. Child Abuse Negl. 2022;134.

422. Thoma D, Strauss F, Mancini L, Gasser T, Jung R, Thoma DS, et al. Minimal invasiveness in soft tissue augmentation at dental implants: A systematic review and meta-analysis of patient-reported outcome measures. Periodontol 2000. 2023;91(1):182-98.

423. Thomas J, Rowe F, Williamson P, Lin E, Thomas J, Rowe F, et al. The effect of leave policies on increasing fertility: a systematic review. Hum Soc Sci Commun. 2022;9(1).

424. Timmer A, Zwanenburg P, Eskes A, Hompes R, Boermeester M, Timmer AS, et al. The Effect of Negative-Pressure Wound Therapy with Instillation Compared to Current Standard Care on Wound Closure Time of Infected Wounds: A Systematic Review and Meta-Analysis. Plast Reconstr Surg. 2022;150(1):176E-88E.

425. Tonning L, O'Brien M, Semciw A, Stewart C, Kemp J, Mechlenburg I, et al. Periacetabular osteotomy to treat hip dysplasia: a systematic review of harms and benefits. Arch Orthop Trauma Surg. 2023;143(6):3637-48.

426. Tonprasong W, Inokoshi M, Shimizubata M, Yamamoto M, Hatano K, Minakuchi S, et al. Impact of direct restorative dental materials on surface root caries treatment. Evidence based and current materials development: A systematic review. Jpn Dent Sci Rev. 2022;58:13-30.

427. Tormen M, Taliento C, Salvioli S, Piccolotti I, Scutiero G, Cappadona R, et al. Effectiveness and safety of COVID-19 vaccine in pregnant women: A systematic review with meta-analysis. Bjog. 2023;130(4):348-57.

428. Triggiani V, Cittadini A, Lisco G, Triggiani V, Cittadini A, Lisco G. Effect of levothyroxine replacement therapy in patients with subclinical hypothyroidism and chronic heart failure: A systematic review. Front Endocrinol. 2022;13.

429. Truong C, Recto C, Lafont C, Canoui-Poitrine F, Belmin J, Lafuente-Lafuente C, et al. Effect of Cholinesterase Inhibitors on Mortality Patients in Patients With Dementia. Neurology. 2022;99(20):E2313-E25.

430. Tsaousi G, Tsitsopoulos P, Foroglou N, Birba V, Tramontana A, Bilotta F, et al. Control of Hemodynamic Responses and Perioperative Outcomes in Transsphenoidal Pituitary Surgery: A Qualitative Systematic Review of the Available Evidence. J Neurosurg Anesthesiol. 2022;34(4):372-83.

431. Tsiropoulos G, Seliniotaki A, Haidich A, Ziakas N, Mataftsi A, Tsiropoulos GN, et al. Comparison of adverse events between intravitreal anti-VEGF and laser photocoagulation for treatment-requiring retinopathy of prematurity: a systematic review. Int Ophthalmol. 2023;43(3):1027-62.

432. Turcotte M, Etherington C, Rowe J, Duong A, Kaur M, Talbot Z, et al. Effectiveness of interprofessional teamwork interventions for improving occupational well-being among perioperative healthcare providers: a systematic review. J Interprofessional Care. 2023;37(6):904-21.

433. Turner H, McManus R, Kiely P, Turner H, McManus R, Kiely P. What Are the Effects of Posterior Corrective Surgery, With or Without Thoracoplasty, on Pulmonary Function in Adolescent Idiopathic Scoliosis? A Systematic Review and Meta-analysis. Glob Spine J. 2023;13(3):910-24.

434. Vaghiri S, Prassas D, Knoefel W, Krieg A, Vaghiri S, Prassas D, et al. The optimal timing of elective surgery in sigmoid diverticular disease: a meta-analysis. Langenbecks Arch Surg. 2022;407(8):3259-74.

435. van de Heyning P, Dazert S, Gavilan J, Lassaletta L, Lorens A, Rajan G, et al. Systematic Literature Review of Hearing Preservation Rates in Cochlear Implantation Associated With Medium- and Longer-Length Flexible Lateral Wall Electrode Arrays. Front Surg. 2022;9.

436. van der Ree M, van Dussen L, Rosenberg N, Stolwijk N, van den Berg S, van der Wel V, et al. Effectiveness and safety of mexiletine in patients at risk for (recurrent) ventricular arrhythmias: a systematic review. Europace. 2022;24(11):1809-23.

437. van Dijk A, Slot A, Portincasa P, Siegerink S, Chargi N, Verstraete C, et al. Systematic review with meta-analysis: Branched-chain amino acid supplementation in liver disease. Eur J Clin Invest. 2023;53(3):e13909.

438. Van Swol J, Myers W, Nguyen S, Eiseman A, Van Swol JM, Myers WK, et al. Revision dacryocystorhinostomy: systematic review and meta-analysis. Orbit. 2023;42(1):1-10.

439. van Weelden W, Birkendahl P, Lalisang R, IntHout J, Kruitwagen R, Romano A, et al. The effect of progestin therapy in advanced and recurrent endometrial cancer: A systematic review and meta-analysis. Bjog. 2023;130(2):143-52.

440. Varma J, Foxall-Smith M, Donovan R, Whitehouse M, Rogers C, Acharya M, et al. Surgical Versus Non-surgical Treatment of Unstable Lateral Compression Type I (LC1) Injuries of the Pelvis With Complete Sacral Fractures in Non-fragility Fracture Patients: A Systematic Review. Cureus J Med Sci. 2022;14(9).

441. Vega-Salas M, Murray C, Nunes R, Hidalgo-Arestegui A, Curi-Quinto K, Penny M, et al. School environments and obesity: a systematic review of interventions and policies among school-age students in Latin America and the Caribbean. Int J Obes. 2023;47(1):5-16.

442. Vermue H, Batailler C, Monk P, Haddad F, Luyckx T, Lustig S, et al. The evolution of robotic systems for total knee arthroplasty, each system must be assessed for its own value: a systematic review of clinical evidence and meta-analysis. Arch Orthop Trauma Surg. 2023;143(6):3369-81.

443. Via R, Bosco F, Giustra F, Lavia A, Artiaco S, Risitano S, et al. Acute Rockwood type III ACJ dislocation: Conservative vs surgical approach. A systematic review and meta-analysis of current concepts in literature. Injury-Int J Care Inj. 2022;53(10):3094-101.

444. Villaman-Santacruz H, Torres-Rosas R, Acevedo-Mascarua A, Argueta-Figueroa L, Villaman-Santacruz H, Torres-Rosas R, et al. Root resorption factors associated with orthodontic treatment with fixed appliances: A systematic review and meta-analysis. Dent Med Probl. 2022;59(3):437-50.

445. Vitkos E, Papadopoulos K, Dimasis P, Weissinger C, Kyrgidis A, Vitkos EN, et al. One miniplate versus two miniplates in the fixation of mandibular angle fractures. An updated systematic review and meta-analysis. J Stomatol Oral Maxillofac Surg. 2022;123(6):E865-E73.

446. Vogel D, Ostermann T, Vogel H, Loskamp K, Fetz K, Vogel D, et al. Recommendation of Neurorehabilitation according to the Padovan-Method Neurofunctional Reorganization (R) for Treating Neurodevelopmental Disorders: A Systematic Review. Complement Med Res. 2022;29(4):330-60.

447. Vogiatzis F, Roussos P, Doulis I, Palikaraki G, Christopoulos P, Sifakakis I, et al. Effects of Surgically Assisted Rapid Palatal Expansion on Facial Soft Tissues: A Systematic Review. Appl Sci-Basel. 2022;12(22).

448. Wagner G, Glechner A, Persad E, Klerings I, Gartlehner G, Moertl D, et al. Risk of Contrast-Associated Acute Kidney Injury in Patients Undergoing Peripheral Angiography with Carbon Dioxide Compared to Iodine-Containing Contrast Agents: A Systematic Review and Meta-Analysis. J Clin Med. 2022;11(23).

449. Wali R, Sacco R, Singh G, Patel V, Wali R, Sacco R, et al. The clinical effect of radiotherapy on pulpal microvasculature: a systematic review. Br Dent J. 2022.

450. Walsh E, Herring M, McMahon J, Walsh EH, Herring MP, McMahon J. A Systematic Review of School-Based Suicide Prevention Interventions for Adolescents, and Intervention and Contextual Factors in Prevention. Prev Sci. 2023;24(2):365-81.

451. Walter N, Rupp M, Olesen U, Alt V, Walter N, Rupp M, et al. Which Pin Site Dressing is the Most Optimal? A Systematic Review on Current Evidence. J Limb Lengthening Reconstr. 2022;8(3):S36-S43.

452. Wang H, Su W, Lowe S, Zhou Z, Bentley R, Zhou Q, et al. Association of Apatinib and Breast Cancer: A systematic review and meta-analysis. Surg Oncol-Oxf. 2022;44.

453. Wang S, Lin X, Guan Y, Huang J, Wang S, Lin X, et al. The clinical outcomes of reni-angiotensin system inhibitors for patients after transcatheter aortic valve replacement: A systematic review and meta-analysis. Front Cardiovasc Med. 2022;9.

454. Wang S, Lyu B, Wang S, Lyu B. Are Current Prophylactic Programs Effective in Preventing Patellar Tendinopathy in Athletes and Recruits? A Meta-Analysis and Trial Sequential Analysis. Sports Health. 2023;15(3):382-5.

455. Wang S, Song J, Zhang H, Wang S, Song J, Zhang H. The effectiveness of social distancing in reducing transmission during influenza epidemics: A systematic review. Public Health Nurs. 2023;40(1):208-17.

456. Wang Z, Su X, Yu Y, Wang Z, Li K, Gao Y, et al. A review of literature and meta-analysis of one-puncture success rate in radiofrequency thermocoagulation with different guidance techniques for trigeminal neuralgia. Eur J Med Res. 2022;27(1).

457. Werter I, Remmelzwaal S, Burchell G, de Gruijl T, Konings I, van der Vliet H, et al. Systemic Therapy for Patients with HER2-Positive Breast Cancer and Brain Metastases: A Systematic Review and Meta-Analysis. Cancers. 2022;14(22).

458. Wetzman A, Lukas C, Gaujoux-Viala C, Mamtani R, Barnetche T, Combe B, et al. Risk of Cancer After Initiation of Targeted Therapies in Patients With Rheumatoid Arthritis and a Prior Cancer: Systematic Review With Meta-Analysis. Arthritis Care Res. 2023;75(2):260-71.

459. Williams M, Hwang B, Huang L, Wilson-Smith A, Brookes J, Eranki A, et al. Robotic versus conventional sternotomy mitral valve surgery: a systematic review and meta-analysis. Ann Cardiothorac Surg. 2022;11(5):490-+.

460. Windle S, Socha P, Nazif-Munoz J, Harper S, Nandi A, Windle SB, et al. The Impact of Cannabis Decriminalization and Legalization on Road Safety Outcomes: A Systematic Review. Am J Prev Med. 2022;63(6):1037-52.

461. Wingfield M, Fini N, Brodtmann A, Williams G, Churilov L, Hayward K, et al. Upper-Limb Motor Intervention Elements That Drive Improvement in Biomarkers and Clinical Measures Post-Stroke: A Systematic Review in a Systems Paradigm. Neurorehabil Neural Repair. 2022;36(10):726-39.

462. Wolf S, Goetz G, Wernly B, Wild C, Wolf S, Goetz G, et al. Subcutaneous implantable cardioverter-defibrillator: a systematic review of comparative effectiveness and safety. ESC Heart Fail. 2023;10(2):808-23.

463. Wolny T, Penas C, Buczek T, Domin M, Granek A, Linek P, et al. The Effects of Physiotherapy in the Treatment of Cubital Tunnel Syndrome: A Systematic Review. J Clin Med. 2022;11(14).

464. Wong J, Pang T, Cheuk N, Liao Y, Bastiampillai T, Chan S, et al. A systematic review on the use of clozapine in treatment of tardive dyskinesia and tardive dystonia in patients with psychiatric disorders. Psychopharmacology. 2022;239(11):3393-420.

465. Wu M, Chang J, Lee Y, Lin P, Tsai T, Wu M-Y, et al. The Effect and Safety of Rapid and Gradual Urinary Decompression in Urine Retention: A Systematic Review and Meta-Analysis. Med Lith. 2022;58(10).

466. Wu Z, Li M, Wang L, Paul A, Raman J, Necchi A, et al. Neoadjuvant systemic therapy in patients undergoing nephroureterectomy for urothelial cancer: a multidisciplinary systematic review and critical analysis. Minerva Urol Nephrol. 2022;74(5):518-27.

467. Xu J, Bai Y, Li E, Xu N, Shi D, Qian J, et al. Efficacy and safety of chemotherapy regimens for first-line treatment of advanced esophageal squamous cell carcinoma in Asia: a systematic review. Expert Rev Anticancer Ther. 2022;22(9):981-98.

468. Xuan G, Ding D, Ning L, Hu Y, Yang F, Tian S, et al. Efficacy and safety of fenofibrate add-on therapy in patients with primary biliary cholangitis refractory to ursodeoxycholic acid: A retrospective study and updated meta-analysis. Front Pharmacol. 2022;13.

469. Yaghi S, Saldanha I, Misquith C, Zaidat B, Shah A, Joudi K, et al. Direct Oral Anticoagulants Versus Vitamin K Antagonists in Cerebral Venous Thrombosis: A Systematic Review and Meta-Analysis. Stroke. 2022;53(10):3014-24.

470. Yan D, Fan H, Chen M, Xia L, Wang S, Dong W, et al. The efficacy and safety of JAK inhibitors for alopecia areata: A systematic review and meta-analysis of prospective studies. Front Pharmacol. 2022;13.

471. Yanagisawa T, Mori K, Quhal F, Kawada T, Mostafaei H, Laukhtina E, et al. Iatrogenic ureteric injury during abdominal or pelvic surgery: a meta-analysis. BJU Int. 2023;131(5):540-52.

472. Yang S, Suh J, Kwon S, Chang M, Yang S, Suh JH, et al. The effect of neurologic music therapy in patients with cerebral palsy: A systematic narrative review. Front Neurol. 2022;13.

473. Yeo B, Koh J, Tan B, Ding Y, Teo Y, Alkan U, et al. Improved Inflammatory and Cardiometabolic Profile After Soft-Tissue Sleep Surgery for Obstructive Sleep Apnea A Systematic Review and Meta-analysis. JAMA Otolaryngol-Head Neck Surg. 2022;148(9):862-9.

474. Yin T, Qin T, Wei K, Shen M, Zhang Z, Wen J, et al. Comparison of safety and effectiveness between laparoscopic and open pancreatoduodenectomy: A systematic review and meta-analysis. Int J Surg. 2022;105.

475. Yu C, Huang T, Tam K, Yu C-Y, Huang T-W, Tam K-W. Management of phyllodes tumor: A systematic review and meta-analysis of real-world evidence. Int J Surg. 2022;107.

476. Yuen H, Tan E, Tran H, Chunilal S, Yuen HLA, Tan E, et al. Idiopathic upper extremity deep vein thrombosis: A systematic review. Eur J Haematol. 2022;109(5):542-58.

477. Yusof R, Norhayati M, Azman Y, Yusof RC, Norhayati MN, Azman YM. Effectiveness of school-based child sexual abuse intervention among school children in the new millennium era: Systematic review and meta-analyses. Front Public Health. 2022;10.

478. Zawistowski M, Nowaczyk J, Domagala P, Zawistowski M, Nowaczyk J, Domagala P. Peritoneal dialysis catheter removal at the time or after kidney transplantation: a systematic review and meta-analysis. Langenbecks Arch Surg. 2022;407(7):2651-62.

479. Zecic F, Smart M, Abbey T, Pazhempallil A, Korban C, Zecic F, et al. Sugammadex-induced anaphylactic reaction: A systematic review. J Anaesth Clin Pharm. 2022;38(3):360-70.

480. Zeraatkar D, Cooper M, Agarwal A, Vernooij R, Leung G, Loniewski K, et al. Long-term and serious harms of medical cannabis and cannabinoids for chronic pain: a systematic review of non-randomised studies. BMJ Open. 2022;12(8).

481. Zhang A, Wang K, Blumenstein K, Brose A, Kemp C, Meister D, et al. For whom and what outcomes does cognitive-behavioral-therapy work among cancer survivors: a systematic review and meta-analysis. Support Care Cancer. 2022;30(11):8625-36.

482. Zhang L, Sun J, Wang K, Zhao H, Zhang X, Ren Z, et al. First- and Second-Line Treatments for Patients with Advanced Hepatocellular Carcinoma in China: A Systematic Review. Curr Oncol. 2022;29(10):7305-26.

483. Zhang Y, Qin Y, Dong P, Ning H, Wang G, Zhang Y, et al. Liver resection, radiofrequency ablation, and radiofrequency ablation combined with transcatheter arterial chemoembolization for very- early- and early-stage hepatocellular carcinoma: A systematic review and Bayesian network meta-analysis for comparison of efficacy. Front Oncol. 2022;12.

484. Zhang Y, Wang J, Fang T, Zhang YW, Wang J, Fang TH. The effect of horticultural therapy on depressive symptoms among the elderly: A systematic review and meta-analysis. Front Public Health. 2022;10.

485. Zhang Z, Chen L, Xu P, Wang Q, Zhang J, Chen K, et al. Effectiveness of automated alerting system compared to usual care for the management of sepsis. npj Digit Med. 2022;5(1).

486. Zhao C, Ye Y, Li Z, Wu X, Zhao C, Hu Z, et al. Effect of goal-directed fluid therapy on renal function in critically ill patients: a systematic review and meta-analysis. Ren Fail. 2022;44(1):777-89.

487. Zhou H, Shen Y, Zhang Z, Liu X, Zhang J, Chen J, et al. Comparison of outcomes of ventral hernia repair using different meshes: a systematic review and network meta-analysis. Hernia. 2022;26(6):1561-71.

488. Zhou J, Lang Y, Wang Z, Gao C, Lv J, Zheng Y, et al. A meta-analysis and systematic review of the effect of loving-kindness and compassion meditations on negative interpersonal attitudes. Curr Psychol. 2023;42:27813-27.

489. Zizzo M, Zanelli M, Sanguedolce F, Palicelli A, Ascani S, Morini A, et al. Gastrectomy with or without Complete Omentectomy for Advanced Gastric Cancer: A Meta-Analysis. Med Lith. 2022;58(9).

490. Zlatanovic P, Jovanovic A, Tripodi P, Davidovic L, Zlatanovic P, Jovanovic A, et al. Chimney vs. Fenestrated Endovascular vs. Open Repair for Juxta/Pararenal Abdominal Aortic Aneurysms: Systematic Review and Network Meta-Analysis of the Medium-Term Results. J Clin Med. 2022;11(22).

491. Zlatanovic P, Jovanovic A, Tripodi P, Davidovic L, Zlatanovic P, Jovanovic A, et al. Chimney Versus Fenestrated Endovascular Versus Open Repair for Juxta/Pararenal Abdominal Aortic Aneurysms: Systematic Review and Network Meta-analysis of the Short-term Results. World JSurg. 2023;47(3):803-23.

492. Zombori-Toth N, Kiss S, Ostarijas E, Alizadeh H, Zombori T, Zombori-Toth N, et al. Adjuvant chemotherapy could improve the survival of pulmonary sarcomatoid carcinoma: A systematic review and meta-analysis. Surg Oncol-Oxf. 2022;44.

**LIST OF EXCLUDED STUDIES**

1. Almohideb M, Almohideb M. Safety and efficacy of nivolumab compared with other regimens in patients with melanoma: A network meta-analysis. Medicine (Baltimore). 2022;101(35).

2. Bhimraj A, Morgan RL, Shumaker AH, Baden LR, Cheng VC, Edwards KM, et al. Infectious Diseases Society of America Guidelines on the Treatment and Management of Patients With COVID-19 (September 2022). Clin Infect Dis. 2024;78(7):e250-e349.

3. Bogossian E, Diosdado A, Barrit S, Al Barajraji M, Annoni F, Schuind S, et al. The Impact of Invasive Brain Oxygen Pressure Guided Therapy on the Outcome of Patients with Traumatic Brain Injury: A Systematic Review and Meta-Analysis. Neurocrit Care. 2022;37(3):779-89.

4. Chen J, Huang Q, Chen W, Lin S, Shi Q, Chen J-m, et al. Clinical Evaluation of Autologous and Allogeneic Stem Cell Therapy for Intrauterine Adhesions: A Systematic Review and Meta-Analysis. Front Immunol. 2022;13.

5. Chen Y, Zhu H, Shen Y, Zhu Y, Sun J, Dai Y, et al. Efficacy and safety of JAK inhibitors in the treatment of alopecia areata in children: a systematic review and meta-analysis. J Dermatol Treat. 2022;33(8):3143-9.

6. Chitturi K, Bhardwaj B, Murtaza G, Karuparthi P, Faza N, Goel S, et al. Clinical Impact of Tricuspid Regurgitation on Transcatheter Edge-to-Edge Mitral Valve Repair for Mitral Regurgitation. Cardiovasc Revascularization Med. 2022;41:1-9.

7. Crookes D, Stanhope K, Suglia S, Crookes DM, Stanhope KK, Suglia SF. Immigrant-Related Policies and the Health Outcomes of Latinx Adults in the United States: A Systematic Review. Epidemiology. 2022;33(4):593-605.

8. Dakis K, Nana P, Brodis A, Kouvelos G, Behrendt C, Giannoukas A, et al. Sarcopenia is a Prognostic Biomarker for Long-Term Survival after Endovascular Aortic Aneurysm Repair: A Systematic Review and Meta-Analysis. Ann Vasc Surg. 2022;83:358-68.

9. Delgado-Ron J, Iroz-Elardo N, Frank L, Delgado-Ron JA, Iroz-Elardo N, Frank L. Health effects of fixed-guideway transit: A systematic review of practice-based evidence. J Transp Health. 2022;26.

10. Dybvik J, Svendsen M, Aune D, Dybvik JS, Svendsen M, Aune D. Vegetarian and vegan diets and the risk of cardiovascular disease, ischemic heart disease and stroke: a systematic review and meta-analysis of prospective cohort studies. Eur J Nutr. 2023;62(1):51-69.

11. Dykukha I, Essner U, Schreiber H, Raithel L, Penner I, Dykukha I, et al. Effects of Sativex? on cognitive function in patients with multiple sclerosis: A systematic review and meta-analysis. Mult Scler Relat Disord. 2022;68.

12. Faulkner L, Howells L, Pepper C, Shaw J, Thomas A, Faulkner LG, et al. The utility of ctDNA in detecting minimal residual disease following curative surgery in colorectal cancer: a systematic review and meta-analysis. Br J Cancer. 2023;128(2):297-309.

13. Fong L, Lee N, Poon J, Chin C, He B, Luo L, et al. Prognostic value of cardiac magnetic resonance derived global longitudinal strain analysis in patients with ischaemic and non-ischaemic dilated cardiomyopathy: a systematic review and meta-analysis. Int J Cardiovasc Imaging. 2022;38(12):2707-21.

14. Freitas T, Ibrahim A, Lourenco A, Chen-Xu J, Freitas T, Ibrahim A, et al. Mortality in COVID-19 patients after proximal femur fracture surgery: a systematic review and meta-analysis. Hip Int. 2023;33(4):762-70.

15. Honeycutt L, Huerne K, Miller A, Wennberg E, Filion K, Grad R, et al. A systematic review of the effects of e-cigarette use on lung function. npj Prim Care Respir Med. 2022;32(1).

16. Hyllestad S, Myrmel M, Lomba J, Jordhoy F, Schipper S, Amato E, et al. Effectiveness of environmental surveillance of SARS-CoV-2 as an early warning system during the first year of the COVID-19 pandemic: a systematic review. J Water Health. 2022;20(8):1223-42.

17. Jafari A, Naghshi S, Shahinfar H, Salehi S, Kiany F, Askari M, et al. Relationship between maternal caffeine and coffee intake and pregnancy loss: A grading of recommendations assessment, development, and evaluation-assessed, dose-response meta-analysis of observational studies. Front Nutr. 2022;9.

18. Keijsers M, Vega-Corredor M, Hoermann S, Tomintz M, Keijsers M, Vega-Corredor MC, et al. Cue Reactivity to Electronic Cigarettes: A Systematic Review. Subst Abus-Res Treat. 2022;16.

19. Kuitunen I, Uimonen M, Haapanen M, Sund R, Helenius I, Ponkilainen V, et al. Incidence of Neonatal Developmental Dysplasia of the Hip and Late Detection Rates Based on Screening Strategy A Systematic Review and Meta-analysis. JAMA Netw Open. 2022;5(8).

20. Leong J, Chen C, Huang C, Cheng S, Chu Y, Chang C, et al. Combination Therapy and Single-Modality Treatment for Acute Low-Tone Hearing Loss: A Meta-Analysis with Trial Sequential Analysis. Brain Sci. 2022;12(7).

21. Leroy R, Bourgeois J, Verleye L, Toma S, Leroy R, Bourgeois J, et al. Should systemic antibiotics be prescribed in periodontal abscesses and pericoronitis? A systematic review of the literature. Eur J Oral Sci. 2022;130(4).

22. Li G, Wang R, Zhang C, Li L, Zhang J, Sun G, et al. Consumption of Non-Nutritive Sweetener during Pregnancy and Weight Gain in Offspring: Evidence from Human Studies. Nutrients. 2022;14(23).

23. Li L, Asemota I, Liu B, Gomez-Valencia J, Lin L, Arif A, et al. AMSTAR 2 appraisal of systematic reviews and meta-analyses in the field of heart failure from high-impact journals. Syst Rev. 2022;11(1).

24. Li Y, Ding Y, Zhao Y, Gui Y, Shen Y, Xiang Q, et al. Prognostic value of soluble urokinase-type plasminogen activator receptor in coronary artery disease: A meta-analysis. Eur J Clin Invest. 2022;52(12).

25. Llamosas-Falcon L, Tran A, Jiang H, Rehm J, Llamosas-Falcon L, Tran A, et al. Liver holidays? A meta-analysis of drinking the same amount of alcohol daily or non-daily and the risk for cirrhosis. Drug Alcohol Rev. 2023;42(1):119-24.

26. Luijken K, van de Wall B, Hooft L, Leenen L, Houwert R, Groenwold R, et al. How to assess applicability and methodological quality of comparative studies of operative interventions in orthopedic trauma surgery. Eur J Trauma Emerg Surg. 2022;48(6):4943-53.

27. Luo Y, Schmidt N, Dubinsky M, Jaffin B, Kayal M, Luo Y, et al. Evaluating lleal Pouch Anal Anastomosis Function: Time to Expand Our ARM-amentarium. Inflamm Bowel Dis. 2023;29(11):1819-25.

28. Magouliotis D, Fergadi M, Zotos P, Rad A, Xanthopoulos A, Bareka M, et al. Differences in long-term survival outcomes after coronary artery bypass grafting using single vs multiple arterial grafts: a meta-analysis with reconstructed time-to-event data and subgroup analyses. Gen Thorac Cardiovasc Surg. 2023;71(2):77-89.

29. Magouliotis D, Zotos P, Fergadi M, Koukousaki D, Zacharoulis D, Diamantis A, et al. Meta-analysis of robot-assisted versus video-assisted McKeown esophagectomy for esophageal cancer. Updates Surg. 2022;74(5):1501-10.

30. Menon J, Struijs F, Whaley P, Menon JML, Struijs F, Whaley P. The methodological rigour of systematic reviews in environmental health. Crit Rev Toxicol. 2022;52(3):167-87.

31. Nedoshivin A, Petrova P, Karpov Y, Nedoshivin A, Petrova PTS, Karpov Y. Efficacy and Safety of Ivabradine in Combination with Beta-Blockers in Patients with Stable Angina Pectoris: A Systematic Review and Meta-analysis. Adv Ther. 2022;39(9):4189-204.

32. Nicholson T, Smith A, McKone E, Gallagher C, Nicholson TT, Smith A, et al. Duration of intravenous antibiotic treatment for acute exacerbations of cystic fibrosis: A systematic review. J Cyst Fibros. 2022;21(4):562-73.

33. Nwaru B, Dierkes J, Ramel A, Arnesen E, Thorisdottir B, Lamberg-Allardt C, et al. Quality of dietary fat and risk of Alzheimer's disease and dementia in adults aged >= 50 years: a systematic review. Food Nutr Res. 2022;66.

34. Palmateer N, Hamill V, Bergenstrom A, Bloomfield H, Gordon L, Stone J, et al. Interventions to prevent HIV and Hepatitis C among people who inject drugs: Latest evidence of effectiveness from a systematic review (2011 to 2020). Int J Drug Policy. 2022;109.

35. Pan Y, Wang S, Liu L, Liu X, Pan Y, Wang S, et al. Whole-gland high-intensity focused ultrasound ablation and transurethral resection of the prostate in the patients with prostate cancer: A systematic review and meta-analysis. Front Oncol. 2022;12.

36. Percival A, Newton C, Mulligan K, Petrella R, Ashe M, Percival A, et al. Systematic review of social prescribing and older adults: where to from here? Fam Med Community Health. 2022;10.

37. Pereira G, Pimentel V, Surita F, Silva A, Brito L, Pereira GMV, et al. Perceived racism or racial discrimination and the risk of adverse obstetric outcomes: a systematic review. Sao Paulo Med J. 2022;140(5):705-18.

38. Ping H, Wen J, Liu Y, Li H, Wang X, Kong X, et al. Unicompartmental knee arthroplasty is associated with lower pain levels but inferior range of motion, compared with high tibial osteotomy: a systematic overview of meta-analyses. J Orthop Surg Res. 2022;17(1).

39. Raheman F, Rojoa D, Hallet C, Yaghmour K, Jeyaparam S, Ahluwalia R, et al. Can Weightbearing Cone-beam CT Reliably Differentiate Between Stable and Unstable Syndesmotic Ankle Injuries? A Systematic Review and Meta-analysis. Clin Orthop Rel Res. 2022;480(8):1547-62.

40. Rajbhoj A, Stroo M, Begnoni G, Willems G, De Llano-Perula M, Rajbhoj AA, et al. Skeletal and soft-tissue changes in humans with untreated normal occlusion throughout lifetime: a systematic review. Odontology. 2023;111(2):263-309.

41. Ribeiro T, Roque F, Ida F, Placido A, Vu M, Hernandez-Munoz J, et al. Early Real-World Data to Assess Benefits and Risks of COVID-19 Vaccines: A Systematic Review of Methods. Vaccines. 2022;10(11).

42. Rokou A, Eleftheriou A, Tsigalou C, Apessos I, Nena E, Dalamaga M, et al. Effect of the Implementation of a Structured Diet Management Plan on the Severity of Obstructive Sleep Apnea: A Systematic Review. Curr Nutr Rep. 2023;12(1):26-38.

43. Sapapsap B, Srisawat C, Suthumpoung P, Luengrungkiat O, Leelakanok N, Saokaew S, et al. Safety of Vitamin K in mechanical heart valve patients with supratherapeutic INR: A systematic review and meta-analysis. Medicine (Baltimore). 2022;101(36).

44. Shlobin N, Wang A, Phillips W, Yan H, Ibrahim G, Elkaim L, et al. Sensorimotor outcomes after resection for perirolandic drug-resistant epilepsy: a systematic review and individual patient data meta-analysis. J Neurosurg-Pediatr. 2022;30(4):410-27.

45. Silva D, Costa F, Baptista I, Santiago T, Lund H, Tarp S, et al. Evidence-Based Research on Effectiveness of Periodontal Treatment in Rheumatoid Arthritis Patients: A Systematic Review and Meta-Analysis. Arthritis Care Res. 2022;74(10):1723-35.

46. Simeone G, Bergamini M, Verga M, Cuomo B, D'Antonio G, Dello Iacono I, et al. Do Vegetarian Diets Provide Adequate Nutrient Intake during Complementary Feeding? A Systematic Review. Nutrients. 2022;14(17).

47. Song Y, Li X, Li C, Xu S, Liu Y, Wu X, et al. What Are the Effects of Electronic Cigarettes on Lung Function Compared to Non-Electronic Cigarettes? A Systematic Analysis. Int J Public Health. 2022;67.

48. Spiga F, Gibson M, Dawson S, Tilling K, Smith G, Munafo M, et al. Tools for assessing quality and risk of bias in Mendelian randomization studies: a systematic review. Int J Epidemiol. 2023;52(1):227-49.

49. Sulis G, Sayood S, Katukoori S, Bollam N, George I, Yaeger L, et al. Exposure to World Health Organization's AWaRe antibiotics and isolation of multidrug resistant bacteria: a systematic review and meta-analysis. Clin Microbiol Infect. 2022;28(9):1193-202.

50. Toubasi A, Al-Sayegh T, Obaid Y, Al-Harasis S, AlRyalat S, Toubasi AA, et al. Efficacy and safety of COVID-19 vaccines: A network meta-analysis. J Evid-Based Med. 2022;15(3):245-62.

51. Ukah U, Aibibula W, Platt R, Dayan N, Reynier P, Filion K, et al. Time-related biases in perinatal pharmacoepidemiology: A systematic review of observational studies. Pharmacoepidemiol Drug Saf. 2022;31(12):1228-41.

52. van Nes K, van Loveren C, Luteijn M, Slot D, van Nes KA, van Loveren C, et al. Health action process approach in oral health behaviour: Target interventions, constructs and groups-A systematic review. Int J Dent Hyg. 2023;21(1):59-76.

53. Varangot-Reille C, Herranz-Gomez A, de la Nava J, Suso-Marti L, Cuenca-Martinez F, Varangot-Reille C, et al. The experience of vertigo: A systematic review of neuroimaging studies. Brain Imaging Behav. 2022;16(6):2797-808.

54. Viswanathan M, Wallace I, Middleton J, Kennedy S, McKeeman J, Hudson K, et al. Screening for Depression and Suicide Risk in Children and Adolescents Updated Evidence Report and Systematic Review for the US Preventive Services Task Force. JAMA-J Am Med Assoc. 2022;328(15):1543-56.

55. Viswanathan M, Wallace I, Middleton J, Kennedy S, McKeeman J, Hudson K, et al. Screening for Anxiety in Children and Adolescents Evidence Report and Systematic Review for the US Preventive Services Task Force. JAMA-J Am Med Assoc. 2022;328(14):1445-55.

56. Wang K, Yu K, Liu P, Lee G, Wong M, Wang K, et al. Can mHealth promotion for parents help to improve their children's oral health? A systematic review. J Dent. 2022;123.

57. Watanabe A, Yasuhara J, Iwagami M, Miyamoto Y, Yamada Y, Suzuki Y, et al. Peripartum Outcomes Associated With COVID-19 Vaccination During Pregnancy A Systematic Review and Meta-analysis. JAMA Pediatr. 2022;176(11):1098-106.

58. Westerhausen R, Westerhausen R. Interhemispheric Integration after Callosotomy: A Meta-Analysis of Poffenberger and Redundant-Target Paradigms. Neuropsychol Rev. 2023;33(4):872-90.

59. Wingrove K, Lawrence M, Machado P, Stephens L, McNaughton S, Wingrove K, et al. Using the Hierarchies of Evidence Applied to Lifestyle Medicine (HEALM) Approach to Assess the Strength of Evidence on Associations between Dietary Patterns and All-Cause Mortality. Nutrients. 2022;14(20).

60. Yuan M, Wu J, Lee J, Cao D, Huynh M, Gallo L, et al. The risk of bias of non-randomized observational studies in deep inferior epigastric perforator flap breast reconstruction: A systematic review using ROBINS-I. J Plast Reconstr Aesthet Surg. 2022;75(11):4096-105.

61. Zhang S, Cui Y, Li J, Tian H, Yun Y, Zhou X, et al. Concomitant transcatheter occlusion versus thoracoscopic surgical clipping for left atrial appendage in patients undergoing ablation for atrial fibrillation: A meta-analysis. Front Cardiovasc Med. 2022;9.

62. Zhu H, Li M, Tian C, Lai H, Zhang Y, Shi J, et al. Efficacy and safety of chinese herbal medicine for treating mild or moderate COVID-19: A systematic review and meta-analysis of randomized controlled trials and observational studies. Front Pharmacol. 2022;13.

63. Zych I, Nasaescu E, Zych I, Nasaescu E. Is radicalization a family issue? A systematic review of family-related risk and protective factors, consequences, and interventions against radicalization. Campbell Syst Rev. 2022;18(3).
